# Isolation and Characterization of Plant-Growth-Promoting Bacteria Associated with *Salvinia auriculata* Aublet

**DOI:** 10.3390/microorganisms12091842

**Published:** 2024-09-06

**Authors:** Jussara Tamires de Souza Silva Goulart, Gabriel Quintanilha-Peixoto, Bruno dos Santos Esteves, Suzane Ariadina de Souza, Pollyanna Santiago Lopes, Nathália Duarte da Silva, Julia Ribeiro Soares, Laura Mathias Barroso, Marina Satika Suzuki, Aline Chaves Intorne

**Affiliations:** 1 Laboratory of Physiology and Biochemistry of Microorganisms, State University of Northern Rio de Janeiro—UENF, Campos dos Goytacazes 28013-602, RJ, Brazil; julliah_@hotmail.com (J.R.S.); laurambarroso@yahoo.com.br (L.M.B.); 2 Laboratory of Function and Chemistry of Proteins and Peptides, State University of Northern Rio de Janeiro—UENF, Campos dos Goytacazes 28013-602, RJ, Brazil; 3 Laboratory of Environmental Sciences (LCA), State University of Northern Rio de Janeiro—UENF, Campos dos Goytacazes 28013-602, RJ, Brazil; brunosnts@yahoo.com.br (B.d.S.E.); marina@uenf.br (M.S.S.); 4 Laboratory of Biotechnology, State University of Northern Rio de Janeiro—UENF, Campos dos Goytacazes 28013-602, RJ, Brazil; 5 Laboratory of Cell and Tissue Biology, State University of Northern Rio de Janeiro—UENF, Campos dos Goytacazes 28013-602, RJ, Brazil; 6 Laboratory of Chemistry and Biology, Instituto Federal de Educação, Ciência e Tecnologia do Rio de Janeiro, Volta Redonda 27213-100, RJ, Brazil

**Keywords:** aquatic plant, plant–bacteria interaction, aquatic environment, biotechnological application, plant growth promotion

## Abstract

*Salvinia auriculata* Aublet is a floating aquatic plant, capable of absorbing the excess of nutrients and water contaminants and can be used in effluent treatment plants. The ability to survive in degraded areas may be related to the association with beneficial bacteria capable of promoting plant growth. However, little is known about the microbiota associated with this aquatic plant and its potential application to the aquatic environment. In this sense, this work aims to identify bacteria associated with *S. auriculata* that could be able to promote plant growth. Eighteen bacterial strains were identified by sequencing of the 16S rRNA gene, belonging to the genera *Agrobacterium*, *Bacillus*, *Curtobacterium*, *Enterobacter*, *Pseudomonas*, *Siccibacter*, and *Stenotrophomonas*. All isolates produced indole compounds, 12 fixed N_2_, and 16 solubilized phosphate. A new strain of *Enterobacter* (sp 3.1.3.0.X.18) was selected for inoculation into *S. auriculata*. For this purpose, 500 mL of nutrient solution and 1 g of the plant were used in the control and inoculated conditions. *Enterobacter* inoculation promoted a significant increase (*p* ≤ 0.05) in fresh plant biomass (17%) after 4 days of cultivation. In summary, the present study characterized 18 plant-growth-promoting bacteria isolated from *S. auriculata* with potential for biotechnological application, such as the production of bioinoculants or biomass resources, to protect or improve plant growth under conditions of stress.

## 1. Introduction

*Salvinia auriculata* Aublet is a free-floating macrophyte in the family Salvinaceae, which, under favorable conditions, quickly grows and spreads by vegetative propagation, colonizing large freshwater surfaces in a short time [1,2]. Due to their small size and ease of cultivation, plants of the genus Salvinia have been used as a model for the study of aquatic macrophytes, including the molecular characterization of these plants at the genomic level [3,4].

Considering that the symbiotic interactions between plants and plant-growth-promoting bacteria (PGPB) in the terrestrial environment are well established, many studies have been carried out to isolate and characterize PGPB associated with important crops [5,6,7]. PGPB stimulate host plant growth through the production of phytohormones, such as indole-3-acetic acid, which directly promote plant growth [8,9,10]. Likewise, PGPB can also improve plant growth through the biological fixation of atmospheric N_2_ (BNF) [11,12] and the solubilization of mineral nutrients such as phosphate, which occurs through the production of organic acids and phosphatases [13,14,15].

Even though microbial interactions with terrestrial plants are relatively well known, the study of PGPB associated with aquatic plants is quite recent. The experiments by Ishizawa [16], for example, showed that there was an increase in the production of plant biomass of the aquatic macrophyte *Lemna minor* when bacterial communities were co-cultivated with the plant. Saha [17] used *Typha angustifolia* as a study model, isolating endophytic microorganisms from this macrophyte. When inoculated in rice, these bacterial isolates also promoted plant growth in the new host. In the work of Ortega-Acosta [18], most of the isolates from *Lemna gibba* were shown to produce indole compounds, which regulate plant growth through various mechanisms [19]. Altogether, these studies show that microbial isolates from aquatic plants might present growth-promoting characteristics, and these PGPB exert positive effects on growth when inoculated into a new host plant, similar to that observed in terrestrial cultures.

In this research, we explore the potential benefits that emerge in studying *S. auriculata* and its associated bacteria as a model. As with most plants, it is hypothesized that *S. auriculata* is colonized by PGPB which might positively shape plant growth. In the present study, we identify bacteria associated with *S. auriculata* capable of promoting plant growth and describe and discuss the PGPB associated with this plant.

## 2. Materials and Methods

### 2.1. Cultivation of Salvinia auriculata Aublet

*Salvinia auriculata* was cultivated in a greenhouse in a nutrient solution at 25% dilution with macronutrients [KNO_3_ (1 M) [VETEC, Duque de Caxias, Brazil], Ca(NO_3_)_2_·4H_2_O (1 M) [Dinâmica, Indaiatuba, Brazil], NH_4_H_2_PO_4_ (1 M) [VETEC], and MgSO_4_·7H_2_O (0.5 M) [Dinâmica]] and micronutrients [KCl (25 mM) [VETEC], H_3_BO_3_ (12.5 mM) [VETEC], MnSO_4_H_2_O (1 mM) [MERCK, Darmstadt, Germany], ZnSO_4_·7H_2_O (1 mM) [Sigma-Aldrich, St. Louis, MO, USA], CuSO_4_·7H_2_O (0.25 mM) [MERCK], H_2_MoO_4_ (0.25 mM) [Dinâmica], FeCl_3_·6H_2_O (53.7 M) [MERCK]]. The solution pH was 5.8 ± 0.1 [20] throughout all assays. According to the National Institute of Meteorology (INMET), with data from the Meteorological Station of Campos dos Goytacazes (RJ, Brazil), the mean photoperiod conditions ranged from 12 h during the day and 12 h at night, with average temperatures of 25 °C during the day and 18 °C at night. The relative humidity of the air was approximately 70%.

### 2.2. Bacterial Strains Isolation and Cultivation

Adult plants with healthy, symptomless, green leaves, with approximately 0.5 cm in leaf diameter and 1 cm in root length, were brought from the greenhouse to the laboratory. Excess water from the plants was removed by placing them for 10 min on paper towels. An amount of 0.5 g of the drained plant tissue was washed in 10 mL of sterile distilled water for 1 min, twice. Then, the plants were transferred to 10 mL of sterile saline solution (0.85% NaCl [Dinâmica]) and incubated for 10 min in ultrasound. Subsequently, the plants were macerated in this same solution and the obtained extract was plated on DYGS medium (2 g/L Anhydrous Dextrose [VETEC], 1.5 g/L Peptone [MERCK], 2 g/L Yeast Extract [KASVI, Pinhais, Brazil], 0.5 g/L K_2_HPO_4_ [MERCK], 0.5 g/L MgSO_4_·7H_2_O [Dinâmica], 1.5 g/L Glutamic Acid [Neon, Suzano, Brazil], 2 g/L Malic Acid [Dinâmica], and 15 g/L Agar [KASVI]) [21]. All materials used for bacterial cultivation were sterilized in a vertical autoclave at 121 °C and 1 atm for 15 min. The plates were incubated at 30 °C for up to 72 h. After the colonies had grown, the bacteria were isolated based on morphological characteristics, including color, elevation (present or absent), edge (regular or irregular), surface (smooth or rough), and optical detail (bright, translucent, or opaque) [22]. The colonies presenting distinct characteristics were incubated in 5 mL liquid DYGS medium at 175 rpm in an orbital shaker at 30 °C for 16 h and the isolates were purified through crossed streaks. The cells were also analyzed under an optical microscope to observe their shape and to analyze their Gram stain pattern.

### 2.3. Molecular Identification of Isolates

Genomic DNA was extracted with Plant DNAzol (Invitrogen, Waltham, MA, USA) according to the manufacturer’s manual. Quantification of genomic DNA was performed using a NanoDrop/2000c Spectrophotometer (Thermo Fisher Scientific, Waltham, MA, USA). For amplification of the 16S rRNA gene, region primers for the Eubacteria domain fD1 (27F 5′AGAGTTTGATCCTGGCTCAG-3′) and rD1 (1429R5′AAGGAGGTGATCCAGCC3′) were used [23]. The amplification reaction contained 2.5 μL of 10× Taq buffer (100 mM Tris-HCl [Invitrogen], pH 8.8, 500 mM KCl [VETEC], 1% Triton-X 100 [MERCK]), 2 μL 25 mM MgCl_2_ (VETEC), 0.5 μL of 10 mM dNTPs, 1 μL of each primer (10 pmol), 0.25 μL of Taq DNA polymerase (2.5 U) (all Invitrogen), 100 ng DNA, and sterile ultrapure water for a final volume of 25 μL. The cycle used was the following: 95 °C for 5 min; 3 pre-amplification cycles of 95 °C for 30 s; and 55 °C for 30 s 72 °C for 2 min. Then, there were another 40 cycles of amplification of 95 °C for 30 s, 55 °C for 30 s, 72 °C for 2 min, and 72 °C for 10 min. In the end, the samples were cooled to 4 °C still in the thermocycler. Sanger sequencing was performed by ACTGene Sequencing Service (Ludwig Biotecnologia Ltd., Alvorada, Rio Grande do Sul, Brazil). Sequence analysis and editing were performed in BioEdit v7.0 [24]. Consensus sequences were identified with the online version of BLASTn (available at https://blast.ncbi.nlm.nih.gov/, accessed on 10 September 2019) [25]. Sequence alignment was performed with Mafft Online (available at https://mafft.cbrc.jp/, accessed on 6 May 2024) [26] and a Maximum Likelihood tree was obtained in MEGA v11 with 500 bootstrap repetitions [27].

### 2.4. Phylogenetic Reconstruction

Other 16S rRNA gene sequences for each given species were obtained from the Nucleotide database of GenBank (available at https://www.ncbi.nlm.nih.gov/nuccore/, accessed on 6 May 2024), using the filter “country = Brazil”. All obtained sequences or a subset of 200 randomly selected sequences (in datasets with over 350 available sequences) were used in an initial alignment with MAFFT [28] also generated a UPGMA phylogenetic tree. This tree was visualized with NCBI’s Tree Viewer (available at https://www.ncbi.nlm.nih.gov/projects/treeview/, accessed on 6 May 2024), and a maximum of 20 tips around our isolates were selected for a refined alignment. The alignment with the selected sequences was also obtained with MAFFT [28], and DNA model selection and phylogeny were obtained with MEGA 11 [29].

### 2.5. Atmospheric N_2_ Fixation

Bacteria were cultivated in DYGS medium until reaching optical density (600 nm) equivalent to OD 1.0 and then centrifuged and washed with saline solution (0.85% NaCl) to remove leftover culture medium and other possible nitrogen sources. An amount of 20 µL aliquots of the culture were inoculated into 10 mL flasks containing 5 mL of the semi-solid culture media NFb and JNFb [30]. Assays were carried out in triplicate and the flasks were kept for 10 days at 30 °C in a bacteriological incubator. After this period, we analyzed the absence or presence of the aerotaxic film formed by the isolates with active nitrogenase enzyme. Cultures were subcultured to new media three times and bacteria that remained forming films were considered positive for BNF.

### 2.6. Production of Indole Compounds

To assess the production of indole compounds, the Salkowsky method [31] was used. The isolates were cultivated until reaching OD (600 nm) = 1.0 and 100 µL culture aliquots were inoculated in 2 mL of DYGS medium, with and without the addition of tryptophan (100 µg·L^−1^) (Synth, Diadema, Brazil), being kept in the dark for 72 at 30 °C in an orbital shaker at 175 rpm. To evaluate the synthesis of indole compounds, 1 mL of the culture was centrifuged at 13,000 rpm for 5 min and 100 µL of the supernatant was transferred to a microplate. Then, 100 µL of Salkowsky’s reagent was added and the plate was incubated in the dark for 30 min. The samples were analyzed in a spectrophotometer at 544 nm and the quantification of indole compounds produced was measured using a calibration curve with indole-3-acetic acid, relating absorbance and concentration of indoles. The assay was performed in triplicate for each isolate.

### 2.7. Phosphate Solubilization

The isolates were cultivated until reaching OD (600 nm) = 1.0. Subsequently, an aliquot of 10 µL of inoculum was placed on a Petri dish containing basal medium (10 g/L Anhydrous Dextrose [VETEC], 5 g/L NH_4_Cl [MERCK], 1 g/L MgSO_4_ [Dinâmica], 1 g/L NaCl [Dinâmica], and 15 g/L Agar [KASVI]) plus 0.8 g·L^−1^ of Ca_3_(PO_4_)_2_ [Dinâmica] [32]. The plates were incubated in an incubator for 72 h and, after this period, the formation of a solubilization halo was observed [32]. The assay was performed in triplicate for each isolate in three independent experiments.

### 2.8. Plant Growth-Promotion Test

Based on the studied plant-growth-promoting characteristics, a bacterium was selected for inoculum in *Salvinia auriculata*. In this sense, plants were acclimated for seven days in a greenhouse using a Hoagland nutrient solution at 25% [33,34]. After this period, samples of plants without disease symptoms, with around 0.5 cm in leaf diameter and 1 cm in root length, were brought to the laboratory, where excess water was removed, and the plants were weighed. Then, 1 g of the plant was transferred to pots containing 500 mL of nutrient solution and 1 mL of selected bacteria inoculum at an OD = 1.0. For the preparation of the inoculum, a loopful of the bacterium from the solid medium was transferred to a 50 mL tube containing 10 mL of DYGS cultivation medium. The tubes were incubated in an orbital shaker for approximately 4 h, and the experiments proceeded once the optical density (OD) = 1.0. No bacterial culture was added to the control condition. The plants were kept in a greenhouse for four days. Fresh and dry biomass were evaluated; for the latter, the plants were placed in an oven at 60 °C for an additional four days, after which the dry biomass of the plants was determined. No bacterial culture was added to the control condition. The plants were kept for four days in a greenhouse.

The photosynthetic pigments (chlorophyll *a*, *b*, and carotenoids) were determined according to the method of [35]. *S. auriculata* plants (50 mg of FB) were placed in plastic test tubes containing 5 mL dimethyl sulfoxide reagent (DMSO) as an organic solvent and kept in the dark. After 4 days, the extract was analyzed in a spectrophotometer at 480 nm, 649 nm, and 665 nm. All the laboratory procedures were carried out in a low-light environment.

### 2.9. Statistical Analysis

Data were tested for adequacy to a normal distribution and for homogeneity of variance using the Shapiro–Wilk and Levene tests, respectively. When necessary, data were log-transformed to ensure homogeneity of variance. A comparison of treatment means was performed using the Student’s *t*-test at a 5% significance level using the in-house R v4 scripts. The data obtained show mean and standard error (SE). Three randomized repetitions were performed, totaling six experimental units.

## 3. Results

### 3.1. Bacteria Associated with Salvinia auriculata Aublet

**Figure 1:**
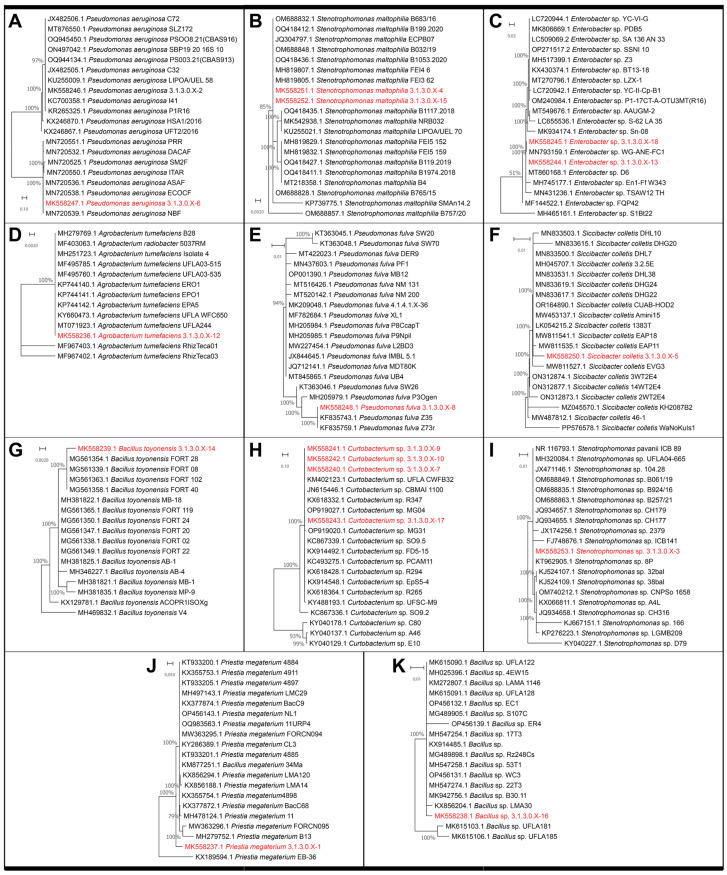
16S rRNA gene Maximum Likelihood phylogenetic distribution of *S. auriculata* isolates. (**A**) *Pseudomonas aeruginosa*. (**B**) *Stenotrophomonas maltophilia*. (**C**) *Enterobacter* sp. (**D**) *Agrobacterium tumefaciens*. (**E**) *Pseudomonas fulva*. (**F**) *Siccibacter coletis*. (**G**) *Bacillus toyonensis*. (**H**) *Curtobacterium* sp. (**I**) *Stenotrophomonas* sp. (**J**) *Priestia megaterium*. (**K**) *Bacillus* sp. Red branched highlight the bacterial strains from this study.

In total, 18 distinct culturable bacteria were isolated from *Salvinia auriculata* Aublet based on the morphological characteristics of their colonies. Description of cell shape and Gram stain were also noted (Table 1). Those 18 isolates were subjected to molecular analysis through partial sequencing of the 16S rRNA gene for taxonomic determination (Table S1, Figure 1). The obtained sequences ranged from 481 to 1479 nucleotides, with an identity coverage of 94% to 99% in BLASTn (Table S1). We could identify seven bacterial genera and at least nine distinct species among these isolates. The 16S rRNA gene sequence was used to obtain a phylogenetic tree of the strains obtained in the isolation, in comparison with other sequences available on GenBank (Figure 1). For most phylogenetic trees, the best DNA substitution model was Jukes–Cantor with uniform substitution rates, except for the phylogenetic trees of *Enterobacter* sp. and *Pseudomonas aeruginosa* (which used the Kimura 2-parameter model with uniform substitution rates) and *Bacillus toyonensis* (which used the Jukes–Cantor model, gamma-distributed rates with invariable sites). For the phylogenetic trees of *Enterobacter* sp. and *Bacillus* sp., 200 sequences were randomly selected for the initial alignment. The full phylogenetic trees in NEXUS format are available in the Supplementary Materials. We could not generate a phylogenetic tree for *Pseudomonas mosselii* due to the small number of available sequences.

### 3.2. Potential of Bacteria to Promote Plant Growth

In our BNF assay (Table 2), 12 bacterial isolates were able to fix atmospheric N_2_, meaning being positive for the formation of an aerotaxic film under the surface of at least one of the two culture media tested for biological N_2_ fixation, which indicates the activity of nitrogenases. The analysis of indole compounds revealed that all isolates were positive for the production of indoles, ranging from 0.70 to 28.18 µg·mL^−1^ (Table 2). The highest production was observed for *Enterobacter* sp. 3.1.3.0.X.18 and the lowest recorded was for *Pseudomonas aeruginosa* 3.1.3.0.X.6. Only two bacteria were unable to solubilize phosphate: *Stenotrophomonas maltophilia* 3.1.3.0.X.15 and the *Curtobacterium* sp. 3.1.3.0.X.17.

#### Growth Promotion of *Salvinia auriculata* by *Enterobacter* sp.

**Figure 2:**
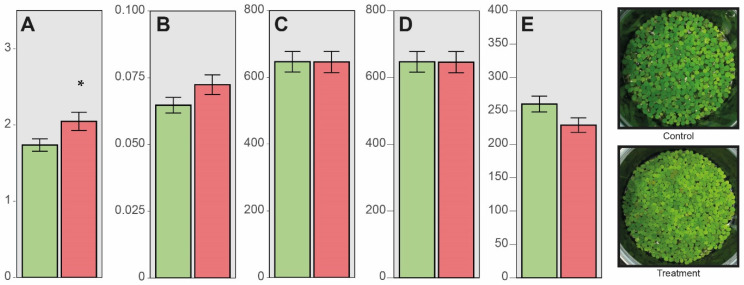
Effect of *Enterobacter* sp. 3.1.3.0.X.18 in *S. auriculata* in plant biomass and photosynthetic pigments. The bacteria promoted an increase in fresh plant biomass (**A**), with no significant difference in dry biomass after 4 days of inoculation (**B**) and in photosynthetic pigments (chlorophyll *a* represented by bar (**C**), chlorophyll *b* represented by bar (**D**), and carotenoids represented by bar (**E**)) according to the *t* Test (*p* ≤ 0.05). Control (only *S. auriculata* plants) represented by green bars. Treatment (plants inoculated with *Enterobacter* sp. 3.1.3.0.X.18 + *S. auriculata*) represented by red bars. The asterisk symbol represents a significant difference between treatments.

The interaction assay with the *Enterobacter* sp. 3.1.3.0.X.18 during 4 days in *Salvinia auriculata* shows a significant difference increase of 17.9% in fresh biomass of inoculated plants compared to the control condition (Figure 2A). There was an increase of 11.9% in dry biomass, but no significant difference (Figure 2B). And, finally, our results show decreases of less than 1.0% in Chlorophyll *a* and Carotenoids, but, on the other hand, it shows a decrease of 12.1% in Chlorophyll *b* (Figure 2C–E). These photosynthetic pigments of inoculated plants compared to the control condition, in summary, do not present statistically significant differences.

## 4. Discussion

The current literature on *Salvinia auriculata* Aublet has described species distribution [35,36] and applications for phytoremediation [33,37,38] that are essential to describe relevant environmental information. Nonetheless, there is still a gap in the knowledge of the association between bacteria and aquatic plants, especially plant-growth-promoting bacteria [39,40]. The present work and some other recent research have brought the isolation of bacteria from aquatic plants and a characterization of growth promoters among the isolated strains. Gilbert [10] isolated 47 endophytic bacteria from *Lemna minor* tissues and evaluated their production of indole compounds. Ishizawa [16] isolated and characterized 22 bacteria from the rhizosphere of *Lemna gibba.* Shehzadi [41] obtained 41 endophytic bacteria isolated from three aquatic plants, finding 8 isolates associated with *Eichhornia crassipes*, 24 with *Typha domingensis*, and 9 with *Pistia stratiotis.* Analysis of the DNA sequences of the 18 isolates revealed that most strains belong to different species and genera. Over the next few paragraphs, we go over the species and traits evaluated in this work, and whether they correspond to a novelty in that species/genus.

Some clades among the species and genera isolated from *S. auriculata* are already known for their growth-promotion traits, like *Curtobacterium* strains, for instance. In the research by Vimal [42] on *Curtobacterium albidum*, SRV4 is described as a diazotrophic and indole compounds producer. Rice plants inoculated with this strain showed significant gains in plant biomass. These data corroborate our results, which are that *Curtobacterium* strains isolated from *S. auriculata* are also diazotrophic and produce indole compounds (Table 2).

*Priestia megaterium* was previously known as *Bacillus megaterium* [43]. The bacterium *P. megaterium* was tested in vitro to produce indole compounds and for phosphate solubilization and showed these characteristics [44]. Chinnaswamy [45] tested growth-promotion traits in *Priestia megaterium,* including a positive indole compound production, but no phosphate solubilization activity or growth in an N-free medium was possible for that strain. In our work, *P. megaterium* 3.1.3.0.X.1 also produced indole compounds and did not fix N_2_, but solubilized phosphate (Table 2). This can be explained by the different cultivation conditions that were used. Meanwhile, Zhao [46] observed the production of indole compounds and phosphate solubilization in this species. Although some *Bacillus* species are recognized as PGPB, the association between *Bacillus toyonensis* and plants is, to the best of our knowledge, a novelty of our research despite the genomic analyses of the species suggesting antimicrobial potential against phytopathogens [47]. Growth-promotion traits in *Agrobacterium tumefaciens* [48] found in the microbiota of *S. auriculata* strain 3.1.3.0.X.12 (this strain was diazotrophic, producing indole compounds and solubilizing phosphate) are also observed elsewhere [49,50]. Banach [49] isolated bacteria from the aquatic plant *Azolla filiculoides* and identified some genera common to those found in *S. auriculata*, such as *Bacillus* and *A. tumefaciens.* Ishizawa [39] examined the colonization and competition dynamics of PGPB and two plant growth inhibitory bacteria inoculated into the aquatic plant *Lemna minor* for seven days. The results showed that PGPB consistently excluded plant growth inhibitors, even though it coexisted almost in the same proportion with another inhibitor strain.

Another species found in the microbiota of *S. auriculata*, *Stenotrophomonas maltophilia*, was able to promote an increase in shoot and root length, chlorophyll content, and total fresh plant biomass when inoculated in tomato plants [51]. The bacterium was identified as an indole producer [52], in the same way as isolates 3.1.3.0.X.4 and 3.1.3.0.X.15. *Siccibacter colletis* was identified in poppy seed isolation. Jackson [53] did not observe the production of indole compounds in their strains. In the present work, strain 3.1.3.0.X.5 is an indole compounds producer, diazotrophic, and phosphate solubilizer. As prementioned, such differences can also result from different growing conditions. *Pseudomonas* is a broad clade, found in various water, soil, and plant environments. These bacteria have high adhesion and biofilm-forming capacities, being used in the production of biosurfactants, in biological control, and as promoters of plant growth [53,54]. In *Pseudomonas aeruginosa*, the production of indole-3-acetic acid and phosphate solubilization have already been verified [55]. Such results agree with the data obtained for the isolates of the genus *Pseudomonas* in the present work (Table 2).

Even though it is largely studied for its clinical aspects, the genus *Enterobacter* is also associated with different plants and soil environments. Some research works describe different species of *Enterobacter* in plants, such as sunflower [56], wheat [57], and corn [58]. These works highlight plant growth-promotion traits that we also evaluated in our research, especially phosphate solubilization and phytohormone production. N_2_-fixating *Enterobacter* strains are also described [59]. Even though these findings corroborate our results (Table 2, Figure 2), this is the first research exploring the association between *Enterobacter* and an aquatic plant, which highlights the relevance of our research.

Photosynthesis is the process by which plants use the energy of sunlight to assimilate CO_2_ from the atmosphere and convert it into soluble carbohydrates, which are then used for plant growth [60]. Makino [61] and Suzuki [40] suggest that an increase in photosynthetic activity associated with increased chlorophyll content is one of the reasons for the growth-promoting effect on aquatic plants. Our results comparing chlorophyll *a*, chlorophyll *b*, and carotenoid production were not statistically significant between *Enterobacter*-inoculated plants and the control. The interaction between aquatic plants and bacteria, although little studied, occurs in water bodies and must have a relevant ecological role in the maintenance of the ecosystem. Plant exudates attract microorganisms, forming true microbial communities with characteristics that can help plant growth, through specific mechanisms and the organic compounds produced, bacteria help to protect the developing plant tissue [62]. Thus, it is possible to envision the application of these PGPBs in contaminated areas together with aquatic plants, seeking an efficient, low-cost, and less costly treatment for the environment.

## 5. Conclusions

Taken together, these results demonstrate that each isolate obtained from *S. auriculata* has the potential to be explored as a biotechnological product in pure cultures or even in microbial consortia, which is more similar to what is found in nature. In the present study, we isolated 18 cultivable bacteria associated with *S. auriculata.* All isolates have shown growth-promotion traits, and plant inoculation with *Enterobacter* sp. 3.1.3.0.X.18 significantly increased the fresh biomass of *S. auriculata* in just four days after inoculation, which reveals the viability of using endogenous PGPB for bioaugmentation, which can be used in processes of remediation.

## Figures and Tables

**Table 1:** Morphological characteristics of colonies and cells, and molecular identification of *S. auriculata* isolates. Nt: nucleotides; Ident: identity. Plus (+) and minus (−) signs indicate positive or negative Gram patterns.

Species	Isolate	GenBank Acc. Code	Length (nt)	Best Hit	Ident (%)	Color	Elevation	Shape	Surface	Optical Property	Gram	Form
*Priestia megaterium*	3.1.3.0.X.1	MK558237	1479	LC606532.1	97.92	Beige	Present	Regular	Smooth	Bright	+	Bacilli
*Pseudomonas aeruginosa*	3.1.3.0.X.2	MK558246	760	OK217196.1	99.17	Green	Absent	Uneven	Rough	Opaque	−	Bacilli
*Stenotrophomonas* sp.	3.1.3.0.X.3	MK558253	818	MH341934.1	97.31	Yellow	Present	Regular	Smooth	Bright	+	Bacilli
*Stenotrophomonas maltophilia*	3.1.3.0.X.4	MK558251	960	CP049956.1	98.09	Yellow	Present	Regular	Smooth	Bright	+	Bacilli
*Siccibacter colletis*	3.1.3.0.X.5	MK558250	1459	NR_134807.1	98.57	White	Present	Uneven	Smooth	Translucent	+	Bacilli
*Pseudomonas aeruginosa*	3.1.3.0.X.6	MK558247	1499	OP677775.1	94.14	Green	Absent	Uneven	Rough	Opaque	−	Bacilli
*Curtobacterium* sp.	3.1.3.0.X.7	MK558240	1449	ON920698.1	94.05	Yellow	Present	Regular	Smooth	Bright	+	Bacilli
*Pseudomonas fulva*	3.1.3.0.X.8	MK558248	1112	KT253977.1	96.82	Yellow	Present	Regular	Smooth	Bright	−	Bacilli
*Curtobacterium* sp.	3.1.3.0.X.9	MK558241	691	MN511778.1	99.40	Yellow	Present	Regular	Smooth	Bright	−	Bacilli
*Curtobacterium* sp.	3.1.3.0.X.10	MK558242	1453	KJ733897.1	96.97	Yellow	Present	Regular	Smooth	Bright	+	Bacilli
*Pseudomonas mosselii*	3.1.3.0.X.11	MK558249	670	KF515676.1	95.17	Green	Present	Regular	Smooth	Bright	+	Bacilli
*Rhizobium radiobacter*	3.1.3.0.X.12	MK558236	1410	MH050420.1	97.69	White	Present	Regular	Smooth	Translucent	−	Bacilli
*Enterobacter* sp.	3.1.3.0.X.13	MK558244	979	KT260465.1	97.32	White	Present	Regular	Smooth	Bright	+	Cocci
*Bacillus toyonensis*	3.1.3.0.X.14	MK558239	1461	MG561363.1	97.96	White	Absent	Uneven	Rough	Opaque	+	Bacilli
*Stenotrophomonas maltophilia*	3.1.3.0.X.15	MK558252	797	MN889390.1	96.60	Yellow	Present	Regular	Smooth	Bright	+	Bacilli
*Bacillus* sp.	3.1.3.0.X.16	MK558238	481	MG461474.1	98.93	Yellow	Present	Regular	Smooth	Bright	+	Bacilli
*Curtobacterium* sp.	3.1.3.0.X.17	MK558243	517	KX618332.1	97.71	Yellow	Present	Regular	Smooth	Bright	+	Bacilli
*Enterobacter* sp.	3.1.3.0.X.18	MK558245	1460	CP118552.1	97.79	White	Present	Regular	Smooth	Translucent	−	Bacilli

**Table 2:** Plant-growth-promoting characteristics in *S. auriculata* Aublet isolates. +: presence of the feature; −: absence of the feature.

Identification	NFb	JNFb	Indoles (µg·mL^−1^)	Solubilization
*P. megaterium* 3.1.3.0.X.1	−	−	2.89	+
*P. aeruginosa* 3.1.3.0.X.2	−	+	24.00	+
*Stenotrophomonas* sp. 3.1.3.0.X.3	+	−	4.70	+
*S. maltophilia* 3.1.3.0.X.4	−	+	6.97	+
*S. colletis* 3.1.3.0.X.5	+	+	7.90	+
*P. aeruginosa* 3.1.3.0.X.6	−	−	0.70	+
*Curtobacterium* sp. 3.1.3.0.X.7	−	−	3.62	+
*P. fulva* 3.1.3.0.X.8	−	+	6.01	+
*Curtobacterium* sp. 3.1.3.0.X.9	−	−	4.81	+
*Curtobacterium* sp. 3.1.3.0.X.10	+	+	1.91	+
*P. mosselii* 3.1.3.0.X.11	+	+	1.44	+
*R. radiobacter* 3.1.3.0.X.12	+	−	19.63	+
*Enterobacter* sp. 3.1.3.0.X.13	−	−	22.72	+
*B. toyonensis* 3.1.3.0.X.14	−	−	1.05	+
*S. maltophilia* 3.1.3.0.X.15	+	+	2.63	−
*Bacillus* sp. 3.1.3.0.X.16	+	+	6.45	+
*Curtobacterium* sp. 3.1.3.0.X.17	−	+	2.66	−
*Enterobacter* sp. 3.1.3.0.X.18	+	+	28.18	+

## Data Availability

All 16S rRNA gene sequences for the 18 isolates presented in this study are available on GenBank under accession codes MK558236-MK558253.

## Supplementary Materials

The following supporting information can be downloaded at: https://www.mdpi.com/article/10.3390/microorganisms12091842/s1, Table S1: Top-five best hits for each bacterial isolate in this study, as presented by online BLASTn.
