# Supplementary material for: Isolation and Characterization of Plant-Growth-Promoting Bacteria Associated with Salvinia auriculata Aublet
Source: Microorganisms. 2024 Sep 6;12(9):1842. doi: 10.3390/microorganisms12091842 (PMC11434440; doi:10.3390/microorganisms12091842)
Supplement: Supplementary file 1 [file microorganisms-12-01842-s001.zip › microorganisms-3029408-supplementary.pdf]

Supplementary Material: Isolation and Characterization of Plant-Growth-Promoting Bacteria Associated with *Salvinia auriculata* Aublet

Jussara Tamires de Souza Silva Goulart, Gabriel Quintanilha-Peixoto, Bruno dos Santos Esteves, Suzane Ariadina de Souza, Pollyanna Santiago Lopes, Nathália Duarte da Silva, Julia Ribeiro Soares, Laura Mathias Barroso, Marina Satika Suzuki and Aline Chaves Intorne

Table S1: Top-five best hits for each bacterial isolate in this study, as presented by online BLASTn.

Sequence	Scientific Name	Accession	Max Score	Total Score	Query Cover (%)	E-value	Ident	Acc. Len
MK558247	Pseudomonas aeruginosa	OP081420.1	2054	2054	92	0.0	94.21	1462
	Pseudomonas aeruginosa	OP677775.1	2050	2050	92	0.0	94.14	1401
	Pseudomonas aeruginosa	KF031122.1	2050	2050	92	0.0	94.14	1407
	Pseudomonas aeruginosa	MK855339.1	2049	2049	92	0.0	94.14	1420
	Pseudomonas aeruginosa	OP142704.1	2049	2049	92	0.0	94.14	1483
MK558237	Priestia aryabhattai	OM910711.1	2414	2414	94	0.0	97.99	1406
	Priestia megaterium	LC606532.1	2410	2410	94	0.0	97.92	1452
	Priestia aryabhattai	OR752014.1	2409	2409	94	0.0	97.92	1392
	Priestia aryabhattai	OR751799.1	2409	2409	94	0.0	97.92	1417
	Bacillus sp.	OR461772.1	2409	2409	94	0.0	97.92	1434
MK558239	Bacillus toyonensis	MG561363.1	2470	2470	97	0.0	97.96	1492
	Bacillus thuringiensis	KY312761.1	2470	2470	97	0.0	97.96	1441
	Bacillus cereus	CP139214.1	2468	34472	97	0.0	97.89	5507140
	Bacillus cereus	OR365310.1	2468	2468	97	0.0	97.89	1474
	Bacillus toyonensis	CP126525.1	2468	34477	97	0.0	97.89	5243919

MK558245	Enterobacter hormaechei	PP754722.1	2436	2436	95	0.0	97.86	1419
	Enterobacter hormaechei	OQ786989.1	2436	2436	95	0.0	97.86	1445
	Enterobacter hormaechei	CP118552.1	2436	19411	95	0.0	97.79	4913168
	Enterobacter hormaechei	MW435394.1	2436	2436	95	0.0	97.86	1436
	Enterobacter sp.	DQ659161.1	2436	2436	95	0.0	97.86	1447
MK558250	Siccibacter colletis	NR_134807.1	2484	2484	96	0.0	98.57	1451
	Siccibacter colletis	OR717480.1	2479	2479	96	0.0	98.50	1469
	Siccibacter colletis	CP134402.1	2479	17194	96	0.0	98.50	4158854
	Siccibacter sp.	OR889460.1	2477	2477	95	0.0	98.57	1404
	Siccibacter colletis	MN833617.1	2468	2468	96	0.0	98.36	1436
MK558242	Curtobacterium subtropicum	OR143696.1	2257	2257	93	0.0	96.97	1522
	Curtobacterium sp.	KJ733897.1	2257	2257	93	0.0	96.97	1369
	Curtobacterium sp.	KJ733885.1	2257	2257	93	0.0	96.97	1372
	Curtobacterium sp.	KJ184963.1	2257	2257	93	0.0	96.97	1428
	Curtobacterium sp.	KJ184962.1	2257	2257	93	0.0	96.97	1427
MK558240	Curtobacterium sp.	ON920698.1	1941	1941	89	0.0	94.05	1415
	Curtobacterium sp.	MT270842.1	1941	1941	89	0.0	94.05	1382
	Curtobacterium sp.	MH769120.1	1940	1940	90	0.0	93.73	1369
	Curtobacterium oceanosedimentum	OR551373.1	1936	1936	89	0.0	93.97	1426
	Curtobacterium pusillum	OQ678260.1	1936	1936	89	0.0	93.97	1363
MK558236	Rhizobium sp.	MK318781.1	2305	2305	95	0.0	97.63	1393
	Agrobacterium pusense	KT380607.1	2305	2305	95	0.0	97.63	1381
	Rhizobium sp.	KM891589.1	2305	2305	95	0.0	97.63	1392
	Agrobacterium pusense	MG997084.1	2303	2303	95	0.0	97.56	1339
	Agrobacterium tumefaciens	MH050420.1	2303	2303	95	0.0	97.69	1352
MK558248	Pseudomonas sp.	KY678888.1	1714	1714	92	0.0	96.82	1396
	Pseudomonas fulva	KT253977.1	1714	1714	92	0.0	96.82	1524

	Pseudomonas sp.	OR570815.1	1714	1714	92	0.0	96.82	1431
	Pseudomonas sp.	OR570812.1	1714	1714	92	0.0	96.82	1435
	Pseudomonas flavescens	OM328051.1	1714	1714	92	0.0	96.82	1396
MK558244	Enterobacter hormaechei	PP660208.1	1365	1365	98	0.0	97.32	941
	Enterobacter sp.	KT260465.1	1365	1365	98	0.0	97.32	1434
	Enterobacter sp.	KJ499995.1	1365	1365	98	0.0	97.32	1515
	Enterobacter hormaechei	KM108494.1	1365	1365	98	0.0	97.32	993
	Enterobacter hormaechei	MW965531.1	1365	1365	98	0.0	97.32	1351
MK558251	Stenotrophomonas maltophilia	PP727400.1	1546	1546	91	0.0	98.09	1402
	Stenotrophomonas sp.	PP094659.1	1546	1546	91	0.0	98.09	1419
	Stenotrophomonas maltophilia	CP139964.1	1546	6175	91	0.0	98.09	5004263
	Stenotrophomonas sp.	CP133161.1	1546	6159	91	0.0	98.09	4493718
	Stenotrophomonas maltophilia	CP133046.1	1546	4607	91	0.0	98.09	4504077
MK558253	Stenotrophomonas sp.	PP094659.1	1428	1428	100	0.0	97.31	1419
	Stenotrophomonas maltophilia	PP086913.1	1428	1428	100	0.0	97.31	1000
	Stenotrophomonas maltophilia	PP086909.1	1428	1428	100	0.0	97.31	1000
	Stenotrophomonas maltophilia	PP086907.1	1428	1428	100	0.0	97.31	1000
	Stenotrophomonas maltophilia	CP139964.1	1428	5710	100	0.0	97.31	5004263
MK558252	Stenotrophomonas sp.	OP811781.1	1362	1362	99	0.0	96.60	1408
	Stenotrophomonas maltophilia	MN889390.1	1360	1360	99	0.0	96.60	1411
	Stenotrophomonas maltophilia	MG905288.1	1360	1360	99	0.0	96.60	1422
	Stenotrophomonas maltophilia	KX350012.1	1360	1360	99	0.0	96.60	1446
	Stenotrophomonas sp.	KT154921.1	1360	1360	99	0.0	96.60	1417
MK558246	Pseudomonas aeruginosa	OK217196.1	1293	1293	94	0.0	99.17	1399
	Pseudomonas aeruginosa	MF599303.1	1291	1291	95	0.0	98.90	1404
	Pseudomonas aeruginosa	OR500522.1	1286	1286	92	0.0	99.72	1405
	Pseudomonas sp.	KF307205.1	1286	1286	92	0.0	99.72	1388

	Pseudomonas aeruginosa	MZ950650.1	1284	1284	91	0.0	99.86	1401
MK558241	Curtobacterium sp.	MN511778.1	1208	1208	96	0.0	99.40	1425
	Curtobacterium sp.	HF563581.1	1208	1208	96	0.0	99.40	1407
	Curtobacterium sp.	AB740341.1	1208	1208	96	0.0	99.40	1111
	Curtobacterium sp.	MW394428.1	1208	1208	96	0.0	99.40	932
	Curtobacterium sp.	MW927018.1	1206	1206	96	0.0	99.40	859
MK558249	Pseudomonas mosselii	KF515676.1	1037	1037	97	0.0	95.17	1433
	Pseudomonas mosselii	CP133092.1	1035	7246	95	0.0	95.81	5702443
	Pseudomonas mosselii	OR230106.1	1035	1035	95	0.0	95.81	696
	Pseudomonas mosselii	OQ682551.1	1035	1035	95	0.0	95.81	1489
	Pseudomonas sp.	OQ651213.1	1035	1035	95	0.0	95.81	730
MK558243	Curtobacterium sp.	KX618332.1	822	822	92	0.0	97.71	1109
	Curtobacterium citreum	GU188922.1	821	821	94	0.0	96.77	1393
	Curtobacterium sp.	MK128452.1	819	819	94	0.0	96.76	1415
	Curtobacterium sp.	KX618262.1	819	819	94	0.0	96.75	1437
	Curtobacterium luteum	OR430490.1	817	817	89	0.0	98.29	1376
MK558238	Bacillus thuringiensis	MG461474.1	832	832	96	0.0	98.93	1498
	Bacillus thuringiensis	KX529821.1	832	832	96	0.0	98.93	1453
	Bacillus thuringiensis	JF899284.1	832	832	97	0.0	98.72	1462
	Bacillus mobilis	OR461808.1	828	828	96	0.0	98.72	1442
	Bacillus toyonensis	OP132950.1	828	828	96	0.0	98.72	1400

NEXUS format early (full) phylogenetic trees

Priestia megaterium

```
#nexus

begin trees;
tree the_tree = ((((1_MK558237_1_3_1_3_0_X-1:0.12599, ((((50_KX355754_1_4898:0, 55_KT933201_1_4885:0):0.01666,
(51_KX355753_1_4911:0, 56_KT933200_1_4884:0):0.01666):0.00352, 53_KT933205_1_4897:0.02018):0.04087,
(54_KT933202_1_4890:0.02266, 120_OQ983563_1_11URP4:0.02266):0.03839):0.06494):0.07462,
((2_KM877251_1_Bacillus_megaterium_34Ma:0.08729, 108_KX856188_1_LMA14:0.08729):0.01367,
106_KX856294_1_LMA120:0.10096):0.09965):0.09092, (((((((((3_KM877248_1_Bacillus_megaterium_31Mb:0.01817,
112_OR472450_1_B3-312:0.01817):0.0065, 79_MW363238_1_FORCN036:0.02468):0.00585, 123_OP456121_1_NC5:0.03052):0.00802,
((((((4_KM877232_1_Bacillus_megaterium_17Mb:0.01793, (6_JQ183034_1_Bacillus_megaterium_P1_4:0.0131,
((7_HQ433239_1_Bacillus_megaterium_CBMAI_1210:0.00996, 44_MG561345_1_FORT_17:0.00996):0.003,
104_KY286396_1_CL13:0.01296):0.00014):0.00482):0.00835, ((((((((((((23_MH497156_1_LMC46:0.00142,
((((((24_MH497151_1_LMC41:0, 25_MH497148_1_LMC36:0):0, 30_MH497140_1_LMC26:0):0, 32_MH497138_1_LMC24:0):0,
38_MH497123_1_LMC08:0):0, 41_MH497119_1_LMC04:0):0.00056, 26_MH497147_1_LMC33:0.00056):0.00086):0.00043,
33_MH497137_1_LMC23:0.00185):0.00022, 40_MH497120_1_LMC05:0.00206):0.00067, 35_MH497135_1_LMC21:0.00273):0.00191,
(37_MH497133_1_LMC18:0.00384, 60_MG778884_1_EP210:0.00384):0.0008):0.00122, 27_MH497144_1_LMC30:0.00586):0.00313,
(((57_MG778897_1_EP224:0.00327, 59_MG778885_1_EP211:0.00327):0.00272, (((48_MG561341_1_FORT_11:0.00322,
61_MG778870_1_EP196:0.00322):1e-05, 70_MW363319_1_FORCN119:0.00323):0.00057,
62_MG778867_1_EP193:0.0038):0.00219):0.00116, 42_MG561352_1_FORT_26:0.00715):0.00184):0.00142,
(((21_MK517695_1_DDEO39:0.00562, (65_MG778858_1_EP184:0.00359, 81_MW363232_1_FORCN030:0.00359):0.00202):0.00021,
71_MW363308_1_FORCN108:0.00582):0.00258, 43_MG561346_1_FORT_18:0.00841):0.00201):0.0019, 99_KY484554_1_UFLA_01-
1011:0.01232):0.00156, 68_MW363330_1_FORCN130:0.01388):0.00208, ((63_MG778865_1_EP191:0.00325,
64_MG778860_1_EP186:0.00325):0.00515, 72_MW363307_1_FORCN107:0.0084):0.00755):0.00314,
36_MH497134_1_LMC19:0.01909):0.00076, 73_MW363298_1_FORCN098:0.01985):0.00643):0.00107,
(((((((((8_KX641548_1_BacI64:0.00324, 20_OR461758_1_P1T01B1:0.00324):0.00307, (((11_OR461791_1_P4T32B1:0.00353,
116_OR466725_1_CNM25:0.00353):0, 114_OR466752_1_CNM35:0.00353):0.00178, 117_OR464524_1_CNM09:0.00531):0.001):0.00085,
((52_KT933208_1_4910:0.00662, (110_KX129778_1_APFSG3isox:0.00413, 113_OR472449_1_B3-311:0.00413):0.00249):0.00014,
((96_MH169333_1_WAB2264:0, 98_MH169257_1_WAB2203:0):0.00107, 97_MH169306_1_WAB2226:0.00107):0.00569):0.0004):0.00084,
(((10_OR461795_1_P6T32B4:0.00379, 18_OR461765_1_P1T01B8:0.00379):0.00032, 77_MW363286_1_FORCN085:0.00411):0.00279,
111_OR472452_1_B3-314:0.00691):0.0011):0.00184, (((((90_MH305351_1_BRM043929:0.00352,
115_OR466749_1_CNM34:0.00352):0.00324, 46_MG561343_1_FORT_15:0.00676):0.00054, 58_MG778887_1_EP213:0.0073):0.00186,
9_OR461796_1_P6T32B5:0.00916):4e-05, 66_MG778776_1_EP85:0.0092):0.00065):0.0025, 78_MW363246_1_FORCN044:0.01234):0.0018,
(100_KX833154_1_IPC7:0.00794, 101_KX833153_1_IPC6:0.00794):0.0062):0.00414, (17_OR461766_1_P1T01B9:0.01163,
12_OR461786_1_P1T32B4:0.01163):0.00665):0.00104, 15_OR461768_1_P2T01B1:0.01932):0.00197,
84_MW363221_1_FORCN019:0.02129):0.00605):0.00501, ((((69_MW363327_1_FORCN127:0.01553,
107_KX856293_1_LMA119:0.01553):0.00362, (82_MW363230_1_FORCN028:0.01686, 122_OP456123_1_EL3:0.01686):0.00229):0.00335,
5_JQ726626_1_Bacillus_megaterium:0.02249):0.00168, ((119_OR464513_1_CNM07:0.01235, 121_OP456143_1_NL1:0.01235):0.00166,
118_OR464523_1_CNM08:0.01402):0.01016):0.00818):0.00156, (124_ON988986_1_1G:0.02376,
```

	125_ON988985_1_2B:0.02376):0.01015):0.002, ((((76_MW363287_1_FORCN086:0.00842, 22_MK517688_1_DDEO32:0.00842):0.00336, 39_MH497121_1_LMC06:0.01178):0.00254, 34_MH497136_1_LMC22:0.01432):0.01075, (29_MH497141_1_LMC27:0.0235, 31_MH497139_1_LMC25:0.0235):0.00157):0.01085):0.00262):0.0082, (((((((((((((13_OR461785_1_P1T32B2:0, 19_OR461760_1_P1T01B3:0):0.00323, 80_MW363237_1_FORCN035:0.00323):0.00329, 87_MK603062_1_7A7-13:0.00653):0.00052, (14_OR461778_1_P6T01B2:0.00646, 47_MG561342_1_FORT_12:0.00646):0.00059):0.00089, 45_MG561344_1_FORT_16:0.00794):0.00149, 92_MH478154_1_28:0.00943):0.00058, 67_OQ268167_1_TS38:0.01001):0.00046, (49_MG561340_1_FORT_10:0.00793, (88_MK598810_1_5A1-13:0.00717, 89_MK598800_1_5C8-13:0.00717):0.00076):0.00253):0.0018, 85_MW363205_1_FORCN003:0.01226):0.00126, 16_OR461767_1_P1T01B10:0.01353):0.00286, (91_MH478155_1_36:0.01565, (94_MH478136_1_32:0.00936, 105_KY286389_1_CL3:0.00936):0.00629):0.00074):0.00223, 93_MH478151_1_35:0.01862):0.00316, 83_MW363227_1_FORCN025:0.02178):0.00497, 75_MW363295_1_FORCN094:0.02675):0.01999):0.00175, 28_MH497143_1_LMC29:0.04848):0.00719, 74_MW363296_1_FORCN095:0.05568):0.01038, (103_KX377872_1_BacC68:0.0419, 95_MH478124_1_11:0.0419):0.02416):0.00238, 102_KX377874_1_BacC9:0.06844):0.04424, 86_MH279752_1_B13:0.11268):0.17885):0.24995, 109_KX189594_1_EB-36:0.54148);<br><br>end;
Bacillus sp.	#nexus<br><br>begin trees;<br>tree the_tree = (((1_MK558238_1_3_1_3_0_X-16:0.06181, ((2_MW116366_1_651F:0.00615, 143_OR472451_1_B3-313:0.00615):0.00043, ((8_OR461798_1_P6T32B7:0.00377, 12_OR461792_1_P5T32B1:0.00377):0.00013, 227_KM272756_1_LAMA_1091:0.0039):0.00268):0.00043, (138_OR656749_1_B1342/15:0.00697, (223_KM272775_1_LAMA_1111:0.00421, 225_KM272766_1_LAMA_1101:0.00421):0.00276):4e-05):0.00012, ((513_KX555404_1:0.00425, 541_KX618298_1_R8:0.00425):0.00255, 520_KX618358_1_R236:0.0068):0.00033):0.00011, (145_OR083081_1_UEA-85:0.00718, (214_KM272810_1_LAMA_1149:0.00541, 219_KM272789_1_LAMA_1127:0.00541):0.00177):7e-05):0.00011, 555_KX618254_1_R72:0.00735):4e-05, ((((243_MW232943_1_BR_10815:0.00029, 245_MW232941_1_BR_10540:0.00029):1e-05, (248_MW232938_1_BR_10532:0, 255_MW232931_1_BR_10441:0):0.00031):0.00032, 257_MW232929_1_BR_10434:0.00063):0.00287, 246_MW232940_1_BR_10537:0.0035):0.00072, 244_MW232942_1_BR_10584:0.00423):0.00316):0.00021, ((487_KY660410_1_UFLA_WFC553:0.00444, 490_KY660407_1_UFLA_WFC549:0.00444):0.00196, 497_KY363405_1_UFLA_ARC094_B13:0.00641):0.00119):0.00043, 517_KX618362_1_R247:0.00803):0.00027, 168_OP456142_1_ER3:0.0083):0.00017, (486_KY660411_1_UFLA_WFC575:0.00671, 488_KY660409_1_UFLA_WFC552:0.00671):0.00176):0.00011, 226_KM272765_1_LAMA_1100:0.00858):0.00017, (187_OM455373_1_MLT8M19:0.00412, 188_OM455372_1_MKC2M3:0.00412):0.00464):0.00043, 484_KY660413_1_UFLA_WFC625:0.00918):0.00027, (((205_KM272844_1_LAMA_1183:0.00568, 417_MH025406_1_7EW37:0.00568):0.00025, (((207_KM272842_1_LAMA_1181:0.00537, 582_MF442279_1_ERR_807:0.00537):6e-05, 419_MH025398_1_5EW21:0.00542):2e-05, 584_MF442277_1_ERR_813:0.00544):0.00049):0.00139, 485_KY660412_1_UFLA_WFC600:0.00732):0.00213):0.00028, (537_KX618305_1_R63:0.00925, ((564_MF442333_1_ERR_673:0.00493, (570_MF442322_1_ERR_701:0.00431, 571_MF442320_1_ERR_705:0.00431):0.00062):0.00123, 572_MF442319_1_ERR_706:0.00616):0.00309):0.00048):0.00023, (((491_KY660406_1_UFLA_WFC548:0.00668, 576_MF442288_1_ERR_785:0.00668):0.00196, 574_MF442317_1_ERR_709:0.00864):0.00096, (578_MF442284_1_ERR_797:0.00904, 585_MF442276_1_ERR_819:0.00904):0.00056):0.00036):0.00021, 526_KX618339_1_R31:0.01017):0.00015, (489_KY660408_1_UFLA_WFC551:0.0097, 581_MF442280_1_ERR_805:0.0097):0.00061):0.00015,

	(206_KM272843_1_LAMA_1182:0.01033, ((558_MF442341_1_ERR_651:0.0052, 561_MF442336_1_ERR_660:0.0052):0.00247,<br>560_MF442337_1_ERR_657:0.00767):0.00266):0.00013):0.00019, ((134_OQ034108_1_MA40:0.00607,<br>559_MF442339_1_ERR_654:0.00607):0.00102, 216_KM272806_1_LAMA_1145:0.00709):0.00356):0.00014,<br>213_KM272825_1_LAMA_1164:0.01079):0.00036, (208_KM272841_1_LAMA_1180:0.00551,<br>542_KX618295_1_R13:0.00551):0.00565):0.00076, (313_MK942746_1_BEM_05:0.00922,<br>580_MF442281_1_ERR_803:0.00922):0.0027):0.00029, (562_MF442335_1_ERR_665:0.008, 604_KY411881_1:0.008):0.00421):0.00066,<br>164_OQ983565_1_58CRP4:0.01287):0.00092, (204_KM272846_1_LAMA_1185:0.01166, 371_MH935519_1_S271:0.01166):0.00213):0.00018,<br>((181_OP456104_1_WR4:0.00768, 406_MH156647_1_13:0.00768):0.00604, 577_MF442285_1_ERR_795:0.01371):0.00025):4e-05,<br>((169_OP456141_1_WR5:0.01166, 174_OP456136_1_NR1:0.01166):0.0013, 496_KY649564_1_UFLA_ARC094:0.01296):0.00104):0.0008,<br>548_KX618283_1_R133:0.01481):0.00065, (185_OP456096_1_WR10:0.01112, 426_MG489908_1_Rz242Cs:0.01112):0.00434):0.00051,<br>(34_MK517706_1_DDEO50:0.01561, 563_MF442334_1_ERR_667:0.01561):0.00037):4e-05, (416_MH025408_1_8EW44:0.01039,<br>418_MH025403_1_6EW28:0.01039):0.00562):0.0005, (135_OQ034104_1_MA32:0.01218,<br>583_MF442278_1_ERR_809:0.01218):0.00434):0.00044, (184_OP456100_1_WR2:0.0169, 463_MG926824_1_CCMICS562:0.0169):6e-<br>05):0.00018, 431_MG489897_1_R115C:0.01714):0.0009, ((((159_ON286898_1_bacteria_LIHM_698:0.01532,<br>(421_MH025393_1_3EW9:0.01276, 423_MH025390_1_1EW2:0.01276):0.00257):0.00064, 414_MH025411_1_10EW49:0.01596):0.00089,<br>683_KX856199_1_LMA25:0.01685):0.00063, (((167_OP456144_1_NC3:0.00992, 565_MF442332_1_ERR_676:0.00992):0.00707,<br>(314_MK942745_1_BEM_04:0.0165, 422_MH025392_1_2EW7:0.0165):0.00049):0.0001, (308_MK942751_1_BEM_12:0.01672,<br>515_KX618415_1_R392:0.01672):0.00037):0.00039):0.00056):0.00066, 424_MG489910_1_Rz247Cs:0.0187):0.0004,<br>183_OP456101_1_NR5:0.0191):0.00067, (((39_MH547272_1_47T1:0.01765, 311_MK942748_1_BEM_08:0.01765):0.00095,<br>((53_MH547257_1_28T2:0.01696, (((((640_KX914655_1:0.0028, 657_KX914523_1:0.0028):0.00028, ((642_KX914647_1:0,<br>652_KX914565_1:0):0.00274, 658_KX914511_1:0.00274):0.00034):0, ((((645_KX914608_1:0, 665_KX914477_1:0):0,<br>671_KX914459_1:0):0.00137, (((663_KX914483_1:0, 666_KX914476_1:0):0, 668_KX914462_1:0):0,<br>669_KX914461_1:0):0.00137):0.0015, (646_KX914599_1:0.00137, ((662_KX914484_1:0, 664_KX914480_1:0):0,<br>670_KX914460_1:0):0.00137):0.0015):0.00021):0.00118, 647_KX914598_1:0.00426):0.0041, (673_KX914448_1:0.00825,<br>674_KX914447_1:0.00825):0.0001):0.00285, 653_KX914550_1:0.01121):0.00575):0.00152, (((222_KM272777_1_LAMA_1113:0.01267,<br>482_KY660415_1_UFLA_WFC772:0.01267):0.00477, (307_MK942752_1_BEM_26:0.01599,<br>316_MK942743_1_BEM_02:0.01599):0.00145):0.00096, (352_MK615098_1_UFLA167:0.01759,<br>428_MG489902_1_R84C:0.01759):0.00082):7e-05):0.00012):0.00095, (((44_MH547266_1_30T2:0.01403,<br>45_MH547265_1_2T2:0.01403):0.00296, (48_MH547262_1_6T3:0.01646, 304_MK942755_1_B30_09:0.01646):0.00052):0.00062,<br>50_MH547260_1_3T4:0.0176):0.00194):0.00022):0.00062, (47_MH547263_1_113T1:0.02004,<br>348_MK615104_1_UFLA182:0.02004):0.00035):0.00014, 568_MF442325_1_ERR_691:0.02052):0.00135,<br>(((((((((((170_OP456140_1_EC3:0.01094, 386_MH093491_1_RG:0.01094):0.00138, (193_OL437041_1_BRM32110:0.00404,<br>212_KM272828_1_LAMA_1167:0.00404):0.00828):0.00011, 501_MF919372_1_64BHI11011:0.01242):0.00113,<br>328_MN099099_1_64NA8:0.01355):0.00179, (286_MT482572_1_ESA_657:0.01266,<br>504_MF919343_1_84BHI1101:0.01266):0.00268):0.00024, (((324_MN099103_1_64BHI1104:0.00812,<br>372_MH093527_1_NEM34:0.00812):0.00566, 566_MF442329_1_ERR_679:0.01379):0.00043, (334_MK759873_1_64R2A10:0.00692,<br>343_MK759863_1_64BHI11010:0.00692):0.0073):0.00137):0.00046, (336_MK759871_1_64R2A4:0.01465,<br>503_MF919344_1_84NA10:0.01465):0.00139):0.00088, 383_MH093494_1_RC:0.01692):0.00104, (325_MN099102_1_64R2A8:0.01368,<br>327_MN099100_1_64NA9:0.01368):0.00428):0.00074, 342_MK759865_1_64BHI11013:0.0187):0.00142, (374_MH093520_1_NEM26:0.01311,<br>(390_MH477670_1_10xB2-11:0.00437, 391_MH470403_1_10xB1-4:0.00437):0.00874):0.00701):0.00101,<br>539_KX618301_1_R141:0.02112):0.00075):0.00084, (546_KX618285_1_R123:0.01147,<br>547_KX618284_1_R131:0.01147):0.01125):0.00074, 144_OR340721_1_CMA12:0.02345):0.00065, 56_MH547253_1_8T2:0.0241):0.00029,

	603_KY411885_1:0.02439):0.00248, 3_KX641578_1_BacI95:0.02687):0.00207, (525_KX618346_1_R128:0.02868,<br>655_KX914544_1:0.02868):0.00027):0.00017, ((36_MH779470_1_Rm1:0.02131, 323_MN121849_1_36S:0.02131):0.00192,<br>166_OP919044_1_MG80:0.02323):0.00589):0.00065, (162_ON286894_1_bacteria_LIHM_690:0.02684,<br>413_MH025413_1_11EW51:0.02684):0.00292):0.00015, (38_MH547274_1_22T3:0.02881,<br>682_KX856204_1_LMA30:0.02881):0.0011):0.0008, 177_OP456131_1_WC3:0.03072):0.00068,<br>430_MG489898_1_Rz248Cs:0.0314):0.00042, (176_OP456132_1_EC1:0.02774, 427_MG489905_1_S107C:0.02774):0.00409):0.00074,<br>(349_MK615103_1_UFLA181:0.03179, 429_MG489899_1_Rz296Ce:0.03179):0.00078):0.0015, (354_MK615091_1_UFLA128:0.02809,<br>425_MG489909_1_Rz273Cm:0.02809):0.00598):0.00126, (171_OP456139_1_ER4:0.03033, 661_KX914485_1:0.03033):0.00499):0.00476,<br>((215_KM272807_1_LAMA_1146:0.02255, 355_MK615090_1_UFLA122:0.02255):0.00041,<br>420_MH025396_1_4EW15:0.02296):0.01713):0.00622, 303_MK942756_1_B30_11:0.0463):0.00294,<br>52_MH547258_1_53T1:0.04924):0.00458, 55_MH547254_1_17T3:0.05382):0.00799):0.00092,<br>347_MK615106_1_UFLA185:0.06273):0.01667, 49_MH547261_1_117T1:0.07939):0;<br><br>end;
Curtobacterium sp.	#nexus<br><br>begin trees;<br>tree the_tree = ((((1_MK558240_1_3_1_3_0_X-7:0.07953, 54_KC867339_1_SO9_5:0.07953):0.17907,<br>(((((((2_MK558241_1_3_1_3_0_X-9:0.05764, 9_OP919027_1_MG04:0.05764):0.01477, (((52_KC493275_1_PCAM11:0.01975,<br>46_KX914492_1_FD5-15:0.01975):0.01354, 55_KC867336_1_SO9_2:0.03329):0.03357,<br>32_KM402123_1_UFLA_CWFB32:0.06686):0.00555):0.01685, (((((((5_ON072232_1_B2811_21:0.00914,<br>15_MG664710_1_61:0.00914):0.00213, (7_OM948663_1_B243_19:0.01006, 8_MH611369_1_ER1/6:0.01006):0.00121):0.01111,<br>((((16_MG664687_1_2:0.00726, 17_MH777911_1_864_2:0.00726):0.00565, 29_MF442342_1_ERR_644:0.01291):0.00201,<br>6_OM948671_1_B2371_21:0.01492):0.00459, 11_OM946590_1_CR1/2022:0.01952):0.00286):0.02078, (28_KX618262_1_R5:0.02586,<br>((30_MF442274_1_ERR_822:0.01412, 48_KP739249_1_4-G:0.01412):0.00091,<br>12_JN615463_1_CBMAI_1118:0.01503):0.01083):0.0173):0.0055, 20_KX618431_1_R320:0.04866):0.0103,<br>(34_KX066800_1_F6D:0.03788, 50_KC493317_1_PCSM53:0.03788):0.02108):0.01234, ((((26_KX618322_1_R254:0.01718,<br>51_KC493313_1_PCSM49:0.01718):0.00422, 13_JN615446_1_CBMAI_1100:0.0214):0.01754, 25_KX618332_1_R347:0.03893):0.02506,<br>33_KY488193_1_UFSC-M9:0.064):0.0073):0.01796):0.0073, 10_OP919020_1_MG31:0.09656):0.02064, ((23_KX618364_1_R265:0.04224,<br>45_KX914548_1_EpS5-4:0.04224):0.03982, 21_KX618428_1_R294:0.08207):0.03513):0.03043, 3_MK558242_1_3_1_3_0_X-<br>10:0.14763):0.04516, 4_MK558243_1_3_1_3_0_X-17:0.19278):0.06582):0.49489, (((((((((((47_KX214243_1_STS7:0.0124,<br>(57_JX471083_1_104_21:0.01141, 59_JX471081_1_104_19:0.01141):0.00099):0.0008, ((27_KX618313_1_R91:0.01125,<br>58_JX471082_1_104_20:0.01125):6e-05, 56_JX471084_1_104_26:0.0113):0.0019):0.007, ((14_MT445053_1_V-58:0.01262,<br>53_KC493265_1_PCAM1:0.01262):0.0024, 49_KC493333_1_PCSM69:0.01502):0.00518):0.00226, (60_JX471080_1_003_38:0.01643,<br>(61_JX471079_1_003_35:0.01522, (18_MG489913_1_R209Ag:0.0103,<br>19_MG489912_1_R208Ag:0.0103):0.00492):0.00121):0.00603):0.05039, 22_KX618426_1_R269:0.07284):0.00757,<br>(((((36_KY040196_1_D20:0, 40_KY040131_1_A34:0):0.00227, 42_KY040109_1_A05:0.00227):0.02141,<br>44_KY040107_1_A03:0.02368):0.01606, 62_GU573532_1_IAB80:0.03975):0.03391, 35_KY040200_1_D32:0.07366):0.00676):0.02226,<br>43_KY040108_1_A04:0.10268):0.01562, (24_KX618336_1_R411:0.11311, 37_KY040180_1_E12:0.11311):0.00519):0.03291,<br>41_KY040129_1_E10:0.15121):0.11819, 39_KY040137_1_A46:0.2694):0.21493, 38_KY040178_1_C80:0.48432):0.26917):0.2584,<br>(31_KM402124_1_UFLA_CWFB33:0.92676, 63_MT996003_1_TGA31:0.92676):0.08513);<br><br>end;

Pseudomonas aeruginosa	#nexus<br><br>begin trees;<br>tree the_tree = ((((((((1_MK558246_1_3_1_3_0_X-2:0.02134, 108_KU255009_1_LIPOA/UEL_58:0.02134):0.02185,<br>113_JX482505_1_C32:0.04319):0.02374, ((26_OQ944134_1_PS003_21_CBAS913_:0.02594, 110_KR265325_1_P1R16:0.02594):0.02303,<br>23_OQ945450_1_PSOO8_21_CBAS916_:0.04897):0.01796):0.02738, 112_JX482506_1_C72:0.09431):0.01411,<br>((((((((4_ON497042_1_SBP19_20_16S_10:0.01687, ((((((9_OP410927_1_BM02:0.0064, (10_OP410926_1_BM01:0.00623,<br>34_OQ613265_1_B1142/18:0.00623):0.00017):0.00156, 30_OQ613269_1_B262/20:0.00797):0.00055,<br>(11_OR041498_1_PS011_22_CBAS987_:0.00507, 14_OQ945459_1_PS019_22_CBAS925_:0.00507):0.00344):0.00167,<br>38_OQ613261_1_B188/18:0.01018):0.00199, ((29_OQ613270_1_B576/20:0.00883, ((47_OQ613252_1_B764/15:0.00314,<br>48_OQ613251_1_B527/15:0.00314):0.00493, 12_OR041497_1_PS007_21_CBAS986_:0.00807):0.00077):0.00165,<br>(16_OQ945457_1_PS017_22_CBAS923_:0.00941, 18_OQ945455_1_PS015_22_CBAS921_:0.00941):0.00108):0.00169):0.00087,<br>((28_OQ944132_1_PS001_21_CBAS911_:0.00894, 115_KF677012_1_C-4:0.00894):0.00156,<br>22_OQ945451_1_PS010_22_CBAS917_:0.0105):0.00255):0.0018, 24_OQ945449_1_PS006_21_CBAS915_:0.01484):0.00203):0.00917,<br>19_OQ945454_1_PS014_22_CBAS920_:0.02604):0.00175, ((((15_OQ945458_1_PS018_22_CBAS924_:0.00526,<br>25_OQ945448_1_PS005_21_CBAS914_:0.00526):0.00255, 20_OQ945453_1_PS013_22_CBAS919_:0.0078):0.00164,<br>21_OQ945452_1_PS012_22_CBAS918_:0.00944):0.0143, (13_OQ945460_1_PS020_22_CBAS926_:0.02028,<br>27_OQ944133_1_PS002_21_CBAS912_:0.02028):0.00347):0.00405):0.0025, 17_OQ945456_1_PS016_22_CBAS922_:0.0303):0.00435,<br>(5_MT876553_1_24LCM:0.02137, 6_MT876552_1_SLZ290:0.02137):0.01328):0.00938, 114_JX482504_1_C15:0.04403):0.01116,<br>((3_ON497043_1_SBP40_18_16S_11:0.03294, (91_KY490643_1_BIB_55:0.01974, 99_KY490622_1_BIB_34:0.01974):0.0132):0.00426,<br>((89_KY490645_1_BIB_58:0.02344, 102_KY490608_1_BIB_20:0.02344):0.00467, (((95_KY490629_1_BIB_41:0.00551,<br>97_KY490625_1_BIB_37:0.00551):0.01187, (((96_KY490626_1_BIB_38:0.00549, 100_KY490621_1_BIB_33:0.00549):0.00463,<br>104_KY490596_1_BIB_07:0.01011):0.00341, 101_KY490620_1_BIB_32:0.01353):0.00385):0.00939, ((90_KY490644_1_BIB_57:0.01234,<br>(93_KY490639_1_BIB_51:0.00367, 98_KY490624_1_BIB_36:0.00367):0.00867):0.00581, (92_KY490642_1_BIB_54:0.0105,<br>(94_KY490630_1_BIB_42:0.00732,<br>103_KY490607_1_BIB_19:0.00732):0.00318):0.00765):0.00862):0.00134):0.00909):0.01799):0.00618,<br>7_MT876551_1_SLZ183:0.06137):0.01294, 8_MT876550_1_SLZ172:0.07431):0.03411):0.04722,<br>(106_KX246870_1_isolate_HSA1/2016:0.07549, 107_KX246867_1_isolate_UFT2/2016:0.07549):0.08014):0.12706,<br>111_KC700358_1_I41:0.2827):0.40798, ((((((((((((((((((31_OQ613268_1_B049/15:0.00472,<br>43_OQ613256_1_B546/16:0.00472):0.00286, 42_OQ613257_1_B825/16:0.00758):0.00094, 88_MG214521_1_TD2_3:0.00851):0.00104,<br>((35_OQ613264_1_B1025/18:0.00381, 44_OQ613255_1_B380/16:0.00381):0.00229, 37_OQ613262_1_B216/18:0.0061):0.00346):0.00294,<br>((((36_OQ613263_1_B284/18:0.00216, 46_OQ613253_1_B1815/15:0.00216):0.00203, 40_OQ613259_1_B587/17:0.0042):0.00167,<br>39_OQ613260_1_B161/18:0.00586):0.00559, (45_OQ613254_1_B056/16:0.01052,<br>41_OQ613258_1_B426/17:0.01052):0.00094):0.00104):0.00553, (32_OQ613267_1_B042/19:0.01519,<br>33_OQ613266_1_B1050/18:0.01519):0.00285):0.00029, (72_MN720535_1_IGAF4:0, 73_MN720534_1_CRF:0):0.01832):0.0065,<br>58_MN720549_1_ITAF:0.02481):0.0089, (62_MN720545_1_SNR:0.02756, 81_MN720526_1_SM2R:0.02756):0.00616):0.0179,<br>(83_MN053857_1_EW48:0.00874, (84_MN053844_1_EW15:0.00174, 85_MN053843_1_EW14:0.00174):0.007):0.04287):0.03166,<br>(((49_MH814624_1_X1107:0.03758, 54_MN720553_1_ESBR:0.03758):0.01639, (((((52_MN720555_1_CATER:0.00918,<br>67_MN720540_1_NBR:0.00918):0.00557, (53_MN720554_1_CATEF:0, 63_MN720544_1_SNF:0):0.01474):0.00916,<br>76_MN720531_1_SBR:0.0239):0.01005, (65_MN720542_1_BACR:0.027, 78_MN720529_1_355F:0.027):0.00695):0.01419,<br>79_MN720528_1_ABR:0.04814):0.00583):0.01494, (74_MN720533_1_DACAR:0.04421,<br>55_MN720552_1_EBLR:0.04421):0.0247):0.01436):0.00414, (50_MN720557_1_VERSBR:0.00991,<br>51_MN720556_1_VERSBF:0.00991):0.0775):0.01822, (66_MN720541_1_BACF:0.06924, 77_MN720530_1_SBF:0.06924):0.03639):0.01393,<br>80_MN720527_1_ABF:0.11955):0.04327, (61_MN720546_1_SM3F:0.15627, 64_MN720543_1_ARAF:0.15627):0.00655):0.0522,<br>(70_MN720537_1_APF:0.124, 60_MN720547_1_SM3R:0.124):0.09102):0.08428, ((59_MN720548_1_AVF:0.02759,<br>109_KT215430_1_P4592:0.02759):0.08684, (86_MH107766_1_LBPMA-EFI:0.02018,<br>105_MG461605_1_CCMICS110:0.02018):0.09424):0.18488):0.22945, 87_MH107114_1_LBPMA-EFII:0.52875):0.16193):0.21989,<br>(((((2_MK558247_1_3_1_3_0_X-6:0.03751, 69_MN720538_1_ECOCF:0.03751):0.08861, 68_MN720539_1_NBF:0.12612):0.04935,

	57_MN720550_1_ITAR:0.17547):0.05513, ((71_MN720536_1_ASAF:0.09364, 82_MN720525_1_SM2F:0.09364):0.01129,<br>75_MN720532_1_DACAF:0.10492):0.12568):0.16392, 56_MN720551_1_PRR:0.39452):0.51604);<br><br>end;
Pseudomonas fulva	#nexus<br><br>begin trees;<br>tree the_tree = (((((((((((((((((((((1_OQ891248_1_T2-MUV:0.0273, 190_KJ667738_1_LV2:0.0273):0.0034,<br>242_KC510008_1_XJ9:0.03069):0.00937, (((283_FJ477107_1_381C:0.01734, 270_HQ220024_1_AKB23:0.01734):0.00617,<br>272_HQ220022_1_AKB21:0.02351):0.00369, 160_KX928043_1_Aa91_R2A02:0.0272):0.01286):0.00512,<br>(22_PP766876_1_kc23711:0.02358, 45_OM979077_1_ChenCCU09:0.02358):0.0216):0.00056, (((((((156_MF421780_1_DBT21:0.01338,<br>43_ON725037_1_Y-722:0.01338):0.00281, 94_MN813905_1_CMSI_115:0.01618):0.0007, 64_MW493204_1_SB2:0.01689):0.00207,<br>20_FJ905911_1_HYTGRFP22:0.01896):0.00321, 82_MN904979_1_2_12:0.02217):0.00199, 50_OK253999_1_HB-3:0.02416):0.00859,<br>((((275_HQ219903_1_ANA72:0.01152, 268_HQ220172_1_SKB2:0.01152):0.00311, 188_JN835514_1_AIMST_4TP6L:0.01463):0.01166,<br>(128_MK544823_1_838_5:0.0162, 140_MK014971_1_KO1M2Aa:0.0162):0.01008):0.00151,<br>155_MF480331_1_BHU_LFM1:0.0278):0.00495):0.00282, 21_PP800466_1_JR88:0.03557):0.01016):0.00335,<br>(((118_MN006193_1_NA213:0.02103, 189_JN835508_1_AIMST_4TP3L:0.02103):0.00748, (52_MZ798463_1_XAM41:0.02234,<br>115_MN030304_1_YOBB02:0.02234):0.00617):0.01188, (((306_AY741159_1_S29:0.01165, 53_MZ798456_1_XAM28:0.01165):0.01158,<br>149_LN995465_1_Pseudomonas_fulva_SR1-65A:0.02323):0.011, (((((159_KT253977_1_OBS-3:0.01118,<br>162_KU991465_1_N14:0.01118):0.00018, 241_KF460530_1_ALEB_41:0.01136):0.0074, 170_KT321277_1_NSMA29:0.01875):0.00534,<br>(284_EU594555_1_2-5:0.01742, 29_OR000434_1_S2:0.01742):0.00667):0.00137, 153_KY938115_1_E6_-<br>_6:0.02545):0.00878):0.00615):0.0087):0.00598, (((((((((((2_MT860293_1_EMR-1:0.00716, 23_PP651544_1_NJB26:0.00716):1e-<br>05, 78_MT994551_1_CUMB_TP-36:0.00716):0.0005, 51_MZ882362_1_FKPF:0.00766):0.00079, 87_MH127734_1_EB277:0.00845):0.00114,<br>((35_OR058584_1_C-D-1:0.00723, ((((((((((((((((((((((((((((((192_KF913772_1_i5_MX:0, 194_KF913770_1_i18_MX:0):0,<br>195_KF913769_1_i30_MX:0):0, 203_KF913761_1_i28_MX:0):0, 204_KF913760_1_i20_MX:0):0, 205_KF913759_1_iA_MX:0):0,<br>206_KF913758_1_iB1_MX:0):0, 207_KF913757_1_i27_MX:0):0, 208_KF913756_1_iJ_MX:0):0, 209_KF913755_1_iK_MX:0):0,<br>210_KF913754_1_iM1_MX:0):0, 211_KF913753_1_i8_MX:0):0, 212_KF913752_1_i3_MX:0):0, 213_KF913751_1_i1_MX:0):0,<br>214_KF913750_1_i29_MX:0):0, 215_KF913749_1_iM2_MX:0):0, 216_KF913748_1_i24_MX:0):0, 217_KF913747_1_i6_MX:0):0,<br>219_KF913745_1_i21_MX:0):0, 220_KF913744_1_iP2_MX:0):0, 222_KF913742_1_i13_MX:0):0, 225_KF913739_1_i11_MX:0):0,<br>226_KF913738_1_iMX48:0):0, 228_KF913736_1_i15_MX:0):0, 221_KF913743_1_i17_MX:0):0.00039,<br>201_KF913763_1_i19_MX:0.00039):0.00017, 202_KF913762_1_i7_MX:0.00057):9e-05, 231_KF913733_1_iMX49:0.00065):9e-05,<br>218_KF913746_1_iP1_MX:0.00074):0.00042, (((((((196_KF913768_1_i26_MX:0, 197_KF913767_1_i16_MX:0):0,<br>198_KF913766_1_i9_MX:0):0, 200_KF913764_1_i25_MX:0):0, 229_KF913735_1_i2_MX:0):9e-05, 199_KF913765_1_i12_MX:9e-<br>05):0.00049, 230_KF913734_1_i4_MX:0.00058):0.00029, (193_KF913771_1_iMX35:0,<br>223_KF913741_1_i14_MX:0):0.00087):0.00029):0.00368, (224_KF913740_1_i23_MX:0.00417,<br>227_KF913737_1_iMX34:0.00417):0.00068):0.00238):0.00107, 90_MN932281_1_Os_Ep_VPA_22:0.0083):0.00129):0.0018,<br>(263_JQ229796_1_1Y1103:0.0108, 102_MN515147_1_Z11:0.0108):0.00059):0.00364, ((((((24_PP528618_1_FYH16:0.00741,<br>146_MH141489_1_AM3:0.00741):0.00169, (28_PP345993_1_S15:0.0058, (46_OM758306_1_TSBB-4:0.0046,<br>101_MN515149_1_Z10:0.0046):0.0012):0.0033):0.0013, (131_MG836000_1_HTI605-2:0.00997, 148_KY968662_1_R-<br>NA2:0.00997):0.00043):0.0001, 267_HQ396916_1_CBMAI_1167:0.0105):0.00052, (30_OR752874_1_YC16:0.00779,<br>(80_MT781362_1_NBRC_16637:0.00535, 255_JX109937_1_QC07:0.00535):0.00244):0.00322):0.00161,<br>((39_OP750200_1_LYX2013112407:0.00862, (145_MH141499_1_E5:0.00739, 147_MH141458_1_R20:0.00739):0.00123):0.0001,<br>(88_MN932309_1_Os_Ep_PPA_24:0.00383, 89_MN932287_1_Os_Ep_VPA_28:0.00383):0.0049):0.00391):0.00102,<br>(163_KU374975_1_AS1258:0.01095, 181_KP234387_1_SW34:0.01095):0.00269):0.00138):0.00199,<br>((((((93_MN082129_1_IILSFPR551:0.00712, 271_HQ220023_1_AKB22:0.00712):0.00441, 25_PP500741_1_HF16:0.01153):0.00143,<br>17_FN599522_1_Pseudomonas_fulva_YMC09/4/B4619:0.01296):0.00068, 31_OR229685_1_NRBM1A:0.01364):0.00061,<br>57_MZ148688_1_UFMG-CM-B225:0.01425):0.00023, 178_KP292606_1_BSW1:0.01448):0.00253):0.00326,

	18_GU244412_1_132:0.02027):0.00183, (((34_OR122185_1_C102:0.01166, 240_KF411350_1_Pseudomonas_fulva:0.01166):0.00193, 182_KP234386_1_SW32:0.0136):0.00247, 299_EU239167_1_KNUC340:0.01607):0.00603):0.01416, 274_HQ219904_1_ANA73:0.03626):0.0058, (185_KM411501_1_KH:0.03403, (56_MZ501281_1_SIEpD3:0.02369, 97_MN813796_1_CMSI_6:0.02369):0.01033):0.00803):0.013):0.00944, ((((((((((((((4_LC507437_1_Pseudomonas_fulva_JCM_11243:0, 5_LC507436_1_Pseudomonas_fulva_JCM_11241:0):0, 70_MW383599_1_BKP_NB20:0):0, 71_MW383598_1_BKP_NB19:0):0, 72_MW383528_1_BKP_NS19:0):0.004, (((125_NR_113857_1_NBRC_16637:0, 264_AB681095_1_Pseudomonas_fulva_NBRC_16639:0):0, 266_AB681093_1_Pseudomonas_fulva_NBRC_16637:0):0.00051, 265_AB681094_1_Pseudomonas_fulva_NBRC_16638:0.00051):0.00349):0.00228, (((6_NR_104280_1_NRIC_0180:0, 13_AB060134_1_Pseudomonas_fulva_AJ_2131:0):0, 14_AB060131_1_Pseudomonas_fulva_AJ_2126:0):0, 15_AB060136_1_Pseudomonas_fulva_NRIC_0180:0):0.00629):0.00195, (60_MW700022_1_CTA23:0.00622, 91_MN932268_1_Os_Ep_VPA_2:0.00622):0.00201):0.00019, (307_AM184228_1_Pseudomonas_fulva_WAB1887:0.00711, (38_OP750219_1_BSY2019072302:0.00328, 92_MN758771_1_JM11:0.00328):0.00383):0.00132):0.00196, (((157_KY511074_1_IN78:0.00482, (252_KC178585_1_Cl-11:0.00186, (304_AM411071_1_Pseudomonas_fulva_Z67zhy:0.00052, 305_AM410620_1_Pseudomonas_fulva_Z58zhy:0.00052):0.00133):0.00296):0.00245, 172_KT580673_1_CanR-83:0.00727):0.00119, 187_EU855189_1_CTSP6:0.00846):0.00193):0.00127, ((62_MW672531_1_JK-G3:0.00367, 253_GU991858_1_2YW4:0.00367):0.00375, 99_MF462916_1_14:0.00742):0.00423):0.00396, 142_MH479103_1_OCC:0.01562):0.00391, (98_MF462953_1_51:0.00936, 138_MK203816_1_T19:0.00936):0.01016):0.01746, 8_KJ561098_1_116FG:0.03699):0.01144, (((((((49_OK415025_1_28_en:0.00913, 176_KP292608_1_BSW8:0.00913):0.00088, 179_KP292605_1_BSW3:0.01001):0.00183, 47_OL966970_1_8:0.01184):0.0018, (66_MW384965_1_144:0.00605, 68_MW384937_1_105:0.00605):0.00759):0.00295, 95_MN813850_1_CMSI_60:0.01659):9e-05, ((67_MW384963_1_142:0.00557, 69_MW384925_1_90:0.00557):0.00191, 164_KP761419_1_SP027:0.00748):0.00921):0.00616, (9_KJ561097_1_A75:0.01544, 10_KJ561096_1_A78:0.01544):0.00741):0.00934, 303_EU215452_1_Pseudomonas_fulva:0.03219):0.01624):0.00827, (((158_KX171072_1_F-4:0.02007, ((232_KF792272_1_faro7_32A:0.01362, 249_KC293832_1_GGRJ12:0.01362):0.00259, (248_KC293855_1_GGRJ35:0.01487, 250_KC293829_1_GGRJ9:0.01487):0.00134):0.00387):0.01314, 247_KC293857_1_GGRJ37:0.03322):0.01517, (288_EF600849_1_BFPB51:0.0413, 291_EF600823_1_BFPB25:0.0413):0.00709):0.0083):0.00781):0.00829, (((3_OQ186745_1_BF:0.04042, 48_OL339359_1_A10:0.04042):0.01021, (114_MK281539_1_SCAU-093:0.04112, 96_MN813829_1_CMSI_39:0.04112):0.00951):0.01947, (289_EF600834_1_BFPB36:0.02908, 171_KR351301_1_ALEB41:0.02908):0.04102):0.00269):0.01373, 33_OR145794_1_Rm6:0.08652):0.02453, ((41_OP001411_1_MB33:0.05654, 61_MW737415_1_1091:0.05654):0.05181, (((286_EF600887_1_BFPB89:0.03314, 290_EF600829_1_BFPB31:0.03314):0.03422, (285_EF600889_1_BFPB91:0.0243, 287_EF600885_1_BFPB87:0.0243):0.04305):0.01155, 292_EF600822_1_BFPB24:0.0789):0.02944):0.0027):0.00874, (161_KU724359_1_Ri1:0.11018, 191_KJ510995_1_AK-33:0.11018):0.00961):0.01461, 136_MK209047_1_4_1_4_0_X-34:0.1344):0.01216, (16_GQ487534_1_Hb-0202:0.08303, 169_KT363044_1_SW8:0.08303):0.06353):0.02095, (234_KF835747_1_Z47:0.13796, 235_KF835744_1_Z36:0.13796):0.02955):0.03721, 73_MW281761_1_L3UC1:0.20471):0.02379, (((152_KY229868_1_P31:0.03608, 276_JN638043_1_Jpyr-4:0.03608):0.05883, ((150_KY273925_1_BIB_1:0.04156, 308_AM161143_1_Pseudomonas_fulva_K3:0.04156):0.03322, 58_MZ067036_1_GTS3_1:0.07479):0.02012):0.04215, (63_MW598162_1_A10:0.06392, 258_JQ830710_1_p96_E05:0.06392):0.07313):0.09145):0.00874, 74_MW281759_1_L2UC1:0.23724):0.1145, (26_PP373775_1_SP2-02:0.02542, ((27_PP346392_1_Ss3-D6:0, 126_MH014944_1_g18:0):0.00717, 122_MH014942_1_g18:0.00717):0.01825):0.32632):0.08095, (59_MZ054390_1_SRCWMB:0.41517, 36_OQ536308_1_KB5:0.41517):0.01752):0.09771, (((((19_GQ848367_1_TNAU_25:0.09451, 37_OQ456213_1_Faraidun_42A6:0.09451):0.01936, 134_MK209049_1_4_1_4_2_X-38:0.11388):0.05536, 254_JX399583_1_E/B/R/P/1:0.16924):0.08197, 75_MW279235_1_L3UB1:0.25121):0.00588, ((((((((((((55_MT125948_1_KUBOTC3:0, 85_MK855418_1_KUBOTAB18:0):0, 124_NR_115610_1_IAM1529:0):0, 312_D84015_1_Pseudomonas_fulva:0):0.01404, 310_AJ516042_1_Pseudomonas_fulva_LE/24:0.01404):0.01063, 137_MK203826_1_T75:0.02467):0.00644, 184_AB495130_1_Pseudomonas_fulva_LB-A:0.03111):0.01135, 311_AJ516041_1_Pseudomonas_fulva_B2/73:0.04246):0.02109, (293_DQ122379_1_iCTE639:0.01969, 294_DQ122353_1_iCTE605:0.01969):0.04385):0.00792, 246_KC250238_1_BAB-2212:0.07146):0.02697, 245_KC250239_1_BAB-2213:0.09843):0.00674, (141_MK014961_1_UI2M1b:0.08213,

	257_JX215557_1_ARB3:0.08213):0.02304):0.04051, 244_KC250246_1_BAB-2221:0.14568):0.08243,<br>295_AY682647_1_HDP20:0.22811):0.02898):0.07012, 173_KT302376_1_UNVERIFIED_ORG__NSA29:0.32721):0.20318):0.23075,<br>((((((((((((((((7_KU672374_1_MRC41:0.00444, 154_KY938087_1_E2_-_4:0.00444):0.00383,<br>277_JN257136_1_SGRAJ09:0.00828):0.00273, (186_KJ958214_1_EX4:0.01051, 117_MN006525_1_L005:0.01051):0.00049):0.00267,<br>((129_MG836070_1_OTI602-2:0, 130_MG836028_1_OTM104-5:0):0, 132_MG835934_1_HTM101-3:0):0.01367):0.00902,<br>(((((((40_OK606012_1_ZM_DL_PA8:0.00326, (273_HQ219982_1_ATY63:0, 280_FJ972539_1_Pseudomonas_fulva_67:0):0.00326):0.00556,<br>77_MW033708_1_CSBB_196:0.00882):0.00137, 133_MK294222_1_CUMB_KMR-01:0.0102):0.00184, ((143_KF717527_1_UF14:0.00792,<br>144_KF717503_1_MP05:0.00792):0.00245, 180_KP234388_1_SW46:0.01037):0.00167):0.004, 175_KP292609_1_BSW9:0.01604):0.00071,<br>183_KJ511900_2_BMB42:0.01675):0.00113, 297_AB248284_1_Pseudomonas_fulva_An_10__=_KMM_3881_:0.01788):0.00482):0.00168,<br>(116_MN006538_1_L020:0.01837, 123_MK548510_1_KY20:0.01837):0.00599):0.00419, (177_KP292607_1_BSW6:0.02392,<br>44_OM568682_1_SMW-566:0.02392):0.00464):0.01187, ((((11_KP684075_1_BS30:0.0094,<br>65_LC441077_1_Pseudomonas_fulva_302:0.0094):0.01137, (((32_OR166219_1_BGRI_EBC_SK23-SW2:0.00754, 309_DQ141541_1_OS-<br>10:0.00754):0.00288, 256_JX109935_1_4C07:0.01042):0.00046, 281_FJ807483_1_TY16:0.01088):0.0099):0.00586,<br>(239_KC634237_1_SR-11:0.01889, 261_JQ618288_1_SMA24:0.01889):0.00774):0.00548,<br>278_JF746160_1_PHA3:0.03211):0.00831):0.00849, 269_HQ220025_1_AKB24:0.04892):0.00998,<br>((((((12_KM654560_1_TTFHRTGL16:0.01334, 302_EU239100_1_KNUC390:0.01334):0.00111, 81_MT634251_1_K3:0.01445):0.00411,<br>(((54_MZ774067_1_AM225:0.00814, (((((103_MN327657_1_YQHJ182:0, 107_MN327652_1_YQHJ181:0):0,<br>113_MN326629_1_YQHJ180:0):0.00319, 174_KP980573_1_JXL21:0.00319):0.00082, (((105_MN327655_1_YQHJ186:0,<br>106_MN327654_1_YQHJ184:0):0, 109_MN327651_1_YQHJ185:0):0, 112_MN326630_1_YQHJ183:0):0.00401):0.00155,<br>(108_MN327653_1_YQHJ188:0, 111_MN326631_1_YQHJ187:0):0.00556):0.00258):0.0007, (104_MN327656_1_YQHJ1810:0,<br>110_MN326632_1_YQHJ189:0):0.00884):0.00116, 300_EU239145_1_KNUC357:0.01):0.00856):0.00158, (139_MG819461_1_AL241:0,<br>298_EU239205_1_KNUC295:0):0.02014):0.0129, ((262_JN835543_1_AIMST_Lce22:0.01187, 296_FJ418772_1_6:0.01187):0.00337,<br>301_EU239144_1_KNUC356:0.01525):0.0178):0.00469, (237_KF835730_1_Z16r:0.02344, (259_JQ614020_1_Pful-2:0.01105,<br>260_JQ614019_1_Pful-1:0.01105):0.01238):0.01429):0.02117):0.00628, (((238_KF835725_1_Z12r:0.01612,<br>282_HM582882_1_AR_PSTS1:0.01612):0.01028, (233_KF835759_1_Z73r:0.01445, 236_KF835743_1_Z35:0.01445):0.01195):0.02936,<br>(166_KT363047_1_SW52:0, 167_KT363046_1_SW26:0):0.05576):0.00942):0.01246, (165_KT363048_1_SW70:0.03725,<br>168_KT363045_1_SW20:0.03725):0.0404):0.03871, (((((42_OP001390_1_MB12:0.01412, 119_MH205985_1_P9Npil:0.01412):0.01793,<br>84_MT516426_1_NM_131:0.03205):0.00679, 100_MN437603_1_PF1:0.03884):0.02495, (((76_MW227454_1_L2BD3:0.03836,<br>251_JQ712141_1_MDT80K:0.03836):0.01084, 120_MH205984_1_P8CcapT:0.0492):0.00632,<br>243_JX844645_1_IMBL_5_1:0.05552):0.00827):0.01368, (151_MF782684_1_XL1:0.06321,<br>121_MH205979_1_P3Ogen:0.06321):0.01426):0.03888):0.01853, 135_MK209048_1_4_1_4_1_X-36:0.13488):0.07659,<br>83_MT520142_1_NM_200:0.21147):0.06348, (127_MK558248_1_3_1_3_0_X-8:0.1691, (79_MT845865_1_UB4:0.10069,<br>86_MT422023_1_isolate_DER9:0.10069):0.0684):0.10585):0.4862):0.23474, 279_AY972290_1_R48:0.99589);<br><br>end;
Siccibacter colletis	#nexus<br><br>begin trees;<br>tree the_tree = (((((((((((((((1_NR_134807_1_1383:0, 2_LK054215_2_1383T:0):0.02831, (22_MN636686_1_BDNA-E86:0.02399,<br>(8_ON312884_1_25WT2E4:0.0179, (9_ON312883_1_18WT2E4:0.01379,<br>11_ON312875_1_4WT2E4:0.01379):0.00411):0.00609):0.00431):0.00194, ((4_OR717480_1_19H1E18:0.01716,<br>24_MT052382_1_C29:0.01716):0.00752, (((((((5_OR431116_1_V163:0.00709, 18_OK103794_1_YSD_YN2:0.00709):0.00137,<br>7_OR150488_1_SSRP30:0.00846):0.00405, 39_MK241859_1_WTB73:0.01251):0.00179, (23_MW033687_1_CSBB_161:0.00973,<br>40_MK241854_1_WTB61:0.00973):0.00457):0.00256, 33_MN833502_1_DHL9:0.01686):0.00301, 34_MN833501_1_DHL8:0.01987):0.00268,<br>36_MN555377_1_N16:0.02255):0.00213):0.00557):0.00353, ((21_MW453137_1_Amini15:0.01072,<br>10_ON312877_1_14WT2E4:0.01072):0.0163, 12_ON312874_1_3WT2E4:0.02701):0.00677):0.00551,<br>29_MN833617_1_DHG22:0.03929):0.00544, 17_MW811527_1_EVG3:0.04474):0.00196, 28_MN833619_1_DHG24:0.0467):0.00672,

	31_MN833531_1_DHL38:0.05342):0.00483, 35_MN833500_1_DHL7:0.05825):0.0026, 30_MN833615_1_DHG20:0.06085):0.00265,<br>32_MN833503_1_DHL10:0.0635):0.03083, 37_MK558250_1_3_1_3_0_X-5:0.09433):0.04182, (((((20_MW487812_1_46-1:0.05715,<br>3_PP576578_1_WaNoKuIs1:0.05715):0.01693, 38_MH045707_1_3_2_5E:0.07408):0.01841, (6_OR164890_1_CUAB-HOD2:0.08447,<br>19_MZ045570_1_KH2087B2:0.08447):0.00802):0.00504, 16_MW811535_1_EAP11:0.09753):0.01971, (15_MW811541_1_EAP18:0.08928,<br>13_ON312873_1_2WT2E4:0.08928):0.02795):0.01892):0.0481, (25_MT052376_1_C7:0.05186,<br>26_MT052374_1_C5:0.05186):0.13239):0.21363, (14_OQ194120_1_TS1-NA3:0.11172, 27_LR736241_1_KS2:0.11172):0.28616);<br><br>end;
Stenotrophomonas maltophilia	#nexus<br><br>begin trees;<br>tree the_tree = (((((1_MK558252_1_3_1_3_0_X-15:0.04063, 59_OM688857_1_B757/20:0.04063):0.01803,<br>(((45_OQ418412_1_B199_2020:0.02953, 32_OQ418427_1_B119_2019:0.02953):0.0095, ((((((((3_MF872196_1_S429:0.01247,<br>(((4_MF079262_1_S431:0.00796, (36_OQ418423_1_B592_2018:0, 70_OM688841_1_B759/17:0):0.00796):0.00255,<br>101_MF443202_1_LGMB149:0.01051):0.00047, (11_OQ955817_1_SM006_22_CBAS932_:0.0075,<br>29_OQ418430_1_B1322_2018:0.0075):0.00347):0.0015):0.00149, (37_OQ418422_1_B427_2021:0,<br>72_OM688839_1_B400/17:0):0.01396):0.00302, ((86_MK543008_1_NRB102:0.0098, ((87_MK543007_1_NRB101:0.00657,<br>88_MK542988_1_NRB081:0.00657):0.00198, 90_MK542978_1_NRB072:0.00855):0.00125):0.00593, ((((((19_OQ418440_1_B748_2020:0,<br>54_OM688862_1_B228/21:0):0.00575, (39_OQ418420_1_B810_2015:0, 75_OM688836_1_B944/16:0):0.00575):0.00202,<br>((22_OQ418437_1_B400_2017:0, 58_OM688858_1_B1142/20:0):0.00449, (33_OQ418426_1_B1807_2018:0,<br>67_OM688844_1_B1106/18:0):0.00449):0.00328):0.00337, (26_OQ418433_1_B1311_2018:0.00794, (52_OM688847_2_B2022/18:0.00054,<br>62_OM688851_1_B157/19:0.00054):0.0074):0.0032):0.00171, ((21_OQ418438_1_B757_2017:0, 57_OM688859_1_B1155/20:0):0.00974,<br>102_MH169256_1_WAB2202:0.00974):0.00311):0.00124, (((30_OQ418429_1_B1549_2015:0, 65_OM688846_1_B1874/18:0):0.00835,<br>79_MT756198_1_IFRO-Sm1:0.00835):0.00334, ((43_OQ418415_1_B316_2016:0, 77_OM688831_1_B142/16:0):0.00876,<br>(49_OM688856_2_B580/20:0, 55_OM688861_1_B187/21:0):0.00876):0.00294):0.00145, ((31_OQ418428_1_B947_2016:0,<br>66_OM688845_1_B1350/18:0):0.01184, (84_MK543114_1_NRB219:0.01074,<br>85_MK543113_1_NRB218:0.01074):0.0011):0.00131):0.00094):0.00164):0.00126):0.00305, ((((((34_OQ418425_1_B1686_2018:0,<br>68_OM688843_1_B473/18:0):0.00293, (35_OQ418424_1_B1370_2018:0, 69_OM688842_1_B888/17:0):0.00293):0.00307,<br>41_OQ418418_1_B1113_2018:0.006):0.00281, (44_OQ418414_1_B0187_2021:0.00792,<br>60_OM688854_1_B179/20:0.00792):0.00089):0.00598, (38_OQ418421_1_B052_2020:0, 74_OM688837_1_B034/17:0):0.01479):0.00094,<br>((25_OQ418434_1_B132_2019:0, 61_OM688853_1_B102/20:0):0.0107, 48_OQ418409_1_B369_2020:0.0107):0.00503):0.0043):0.00146,<br>107_MG685766_1_JN40:0.0215):0.00114, (112_KC622052_1_STM11:0.01257, 116_JQ304797_1_ECPB07:0.01257):0.01007):0.00237,<br>((47_OQ418410_1_B8541_2014:0, 78_OM688828_1_B765/15:0):0.01821, (42_OQ418416_1_B475_2018:0,<br>76_OM688832_1_B683/16:0):0.01821):0.0068):0.00632, (((((28_OQ418431_1_B1385_2018:0, 64_OM688848_1_B032/19:0):0.01272,<br>23_OQ418436_1_B1053_2020:0.01272):0.0028, (24_OQ418435_1_B1117_2018:0.0138,<br>111_KU255021_1_LIPOA/UEL_70:0.0138):0.00172):0.00817, 82_MT218358_1_B4:0.02369):0.00417,<br>46_OQ418411_1_B1974_2018:0.02786):0.00348):0.0077):0.00708, 91_MK542938_1_NRB032:0.04612):0.01255):0.02339,<br>(2_MK558251_1_3_1_3_0_X-4:0.02539, ((92_MH819832_1_FEI5_159:0, 93_MH819829_1_FEI5_152:0):0.00409,<br>((((((94_MH819815_1_FEI4_108:0, 95_MH819814_1_FEI4_107:0):0, 96_MH819812_1_FEI4_92:0):0, 97_MH819810_1_FEI4_49:0):0,<br>98_MH819808_1_FEI4_13:0):0, 99_MH819807_1_FEI4_6:0):0, 100_MH819805_1_FEI3_62:0):0.00409):0.02131):0.05667):0.08489,<br>6_KP739775_1_SMAn14_2:0.16695):0.50042, ((((((((((18_OQ418441_1_B843_2017:0.00474,<br>40_OQ418419_1_B1140_2018:0.00474):0.00626, 27_OQ418432_1_B1689_2018:0.011):0.00386, (((53_OM688827_2_B759/15:0.00413,<br>71_OM688840_1_B407/17:0.00413):0.00572, 51_OM688852_2_B770/19:0.00985):0.00361,<br>63_OM688850_1_B129/19:0.01346):0.0014):0.00402, 110_KU180334_1_LEM12:0.01887):0.00408, ((20_OQ418439_1_B368_2020:0,<br>56_OM688860_1_B0105/21:0):0.01869, ((50_OM688855_2_B203/20:0, 73_OM688838_1_B098/17:0):0.01541,<br>81_JN615424_1_CBMAI_1076:0.01541):0.00328):0.00427):0.00849, ((((9_OQ955819_1_SM009_22_CBAS934_:0.00938,<br>14_OQ955814_1_SM003_21_CBAS929_:0.00938):0.00226, 12_OQ955816_1_SM005_22_CBAS931_:0.01165):0.00173,

	(13_OQ955815_1_SM004_22_CBAS930_:0.00826, (15_OQ955813_1_SM002_21_CBAS928_:0.00566, 16_OQ955812_1_SM001_21_CBAS927_:0.00566):0.0026):0.00511):0.00914, 108_KX856268_1_LMA94:0.02251):0.00893):0.00855, ((83_MK543118_1_NRB225:0.02175, 89_MK542985_1_NRB078:0.02175):0.00549, 114_JQ183019_1_SG40:0.02724):0.01275):0.00569, 115_JQ183018_1_SG59:0.04568):0.03842, (109_KX760142_1_GB701:0.06028, 113_KC113607_1_UFLA03-259:0.06028):0.02382):0.04265, (((103_KY490631_1_BIB_43:0.02547, 106_KY490594_1_BIB_05:0.02547):0.01949, (104_KY490623_1_BIB_35:0.01421, 105_KY490605_1_BIB_16:0.01421):0.03076):0.02302, (8_OQ955820_1_SM010_22_CBAS935_:0.05, 80_MH279790_1_B8:0.05):0.01798):0.05877):0.13333, (((5_KP739776_1_SMAn14_3:0.03314, 7_KP739774_1_SMAn14_1:0.03314):0.06556, 10_OQ955818_1_SM008_22_CBAS933_:0.09871):0.14463, 17_OQ326573_1_BRM_063574:0.24334):0.01674):0.4073);<br><br>end;
Stenotrophomonas sp.	#nexus<br><br>begin trees;<br>tree the_tree = ((((((((((1_MK558253_1_3_1_3_0_X-3:0.05856, 207_FJ748676_1_SICB141:0.05856):0.07305, ((((((((((138_JX174274_1_S2397:0.00929, 145_JX174202_1_S2325:0.00929):0.00119, 146_JX174201_1_S2324:0.01048):0.00459, 109_KC493334_1_SPCSM70:0.01507):0.00318, (142_JX174241_1_S2364:0.01378, 112_KC493290_1_SPCAM26:0.01378):0.00447):0.00528, (((42_MF326469_1_131S:0.00795, 44_MG489914_1_Rz291Ce:0.00795):0.00343, 141_JX174242_1_S2365:0.01138):0.00509, (139_JX174257_1_S2380:0.01613, 46_MG685711_1_clone_F5aug:0.01613):0.00034):0.00706):0.00471, ((134_JQ934656_1_SCH203:0.0013, 136_JQ934654_1_SCH17:0.0013):0.00461, 135_JQ934655_1_SCH177:0.00592):0.02233):0.01189, ((((((7_OM688863_1_B257/21:0.00474, 9_OM688835_1_B924/16:0.00474):0.00626, 8_OM688849_1_B061/19:0.011):0.00222, 131_JX471146_1_S104_28:0.01322):0.00151, 33_MH320084_1_UFLA04-665:0.01473):0.00263, (210_NR_116793_1_Stenotrophomonas_pavanii_strain_ICB_89_16S_ribosomal_RNA__partial_sequence:0, 211_FJ748683_2_Stenotrophomonas_pavanii_strain_ICB_89:0):0.01736):0.00813, 63_KX066811_1_A4L:0.02548):0.00428, (132_JQ934658_1_SCH316:0.01104, 133_JQ934657_1_SCH179:0.01104):0.01872):0.01038):0.02558, 100_KJ667151_1_S166:0.06572):0.0172, 105_KP276223_1_SLGMB209:0.08292):0.02077, ((140_JX174256_1_S2379:0.02401, (6_OM740212_1_CNPSo_1658:0.01066, (125_KJ524109_1_S38bal:0.00391, 126_KJ524107_1_S32bal:0.00391):0.00674):0.01335):0.01529, 103_KT962905_1_S8P:0.0393):0.0644):0.02792):0.08542, 64_KY040227_1_D79:0.21703):0.0097, 206_FJ748677_1_SICB134:0.22673):0.27921, 31_KY229230_1_UNVERIFIED__UAGC_925:0.50594):0.27819, ((((((38_MH426878_1_BW3-4:0.17002, 101_KX170984_1_Uncultured_Sclone_Cu_94:0.17002):0.04563, 35_MH454624_1_CW2-7:0.21565):0.07345, 39_MH426906_1_MB2-6:0.2891):0.03743, 36_MH426883_1_BW2-11:0.32653):0.10206, (200_JF303693_1_S4PR:0.30932, 201_FJ748682_1_SICB91:0.30932):0.11927):0.07013, (40_MH426886_1_MW2-5:0.01046, 41_MH424483_1_BW2-12:0.01046):0.48826):0.12498, 37_MH426879_1_BW3-9:0.62369):0.16044):0.16043, ((((((((((((((((((2_OQ034126_1_MA65:0.02691, 62_KX066819_1_C2L:0.02691):0.01224, (174_JX842802_1_SE11:0.02661, 171_JX842806_1_SEE3:0.02661):0.01255):0.00444, ((189_JX842784_1_SCE5:0.02461, 191_JX842782_1_SCE2:0.02461):0.00992, 190_JX842783_1_SCE4:0.03453):0.00907):0.01735, ((((177_JX842798_1_SE7:0.0161, 106_KT189486_1_SDsF_N016:0.0161):0.00596, 115_KF878282_1_SESA_0008:0.02206):0.01439, 26_MK424600_1_ESA_399:0.03645):0.02124, ((((11_OM688830_1_B102/16:0.01535, 104_KX376306_1_SCFA2_1:0.01535):0.00133, 61_KY452400_1_P98:0.01668):0.00391, 182_JX842792_1_SE1:0.02059):0.01839, (108_KC493341_1_SPCSM77:0.03067, 51_MF442298_1_ERR_761:0.03067):0.00831):0.01872):0.00326):0.00533, ((((116_KJ524267_1_Shata96:0, 118_KJ524226_1_Shata49:0):0, 119_KJ524222_1_Shata43:0):0, 120_KJ524221_1_Shata42:0):0.01728, ((121_KJ524192_1_Sacu96:0, 123_KJ524159_1_Sacu46:0):0, 124_KJ524156_1_Sacu40:0):0.01728):0.049):0.00776, 107_KT189456_1_SDsA_N028:0.07404):0.00713, ((((((((((32_MK321682_1_CCMICS_531:0.01883, 152_JX842834_1_SNOE7:0.01883):0.00197, 154_JX842830_1_SNOE3:0.0208):0.00413, 111_KC493303_1_SPCSM39:0.02492):0.0038, (179_JX842796_1_SE5:0.02106, 151_JX842835_1_SNOE8:0.02106):0.00767):0.0078, 181_JX842793_1_SE2:0.03653):0.01093, ((((((((((((((((((((((66_KX914642_1_EpD2-11:0, 68_KX914633_1_EpD2-22:0):0, 71_KX914627_1_EpD2-8:0):0, 76_KX914613_1_EpD4-10:0):0, 86_KX914529_1_FD2-14:0):0, 87_KX914528_1_FD2-15:0):0, 88_KX914527_1_FD2-17:0):0, 89_KX914512_1_FD4-13:0):0, 90_KX914497_1_FD5-10:0):0, 91_KX914496_1_FD5-11:0):0,

	92_KX914489_1_FD5-19:0):0, 94_KX914487_1_FD5-21:0):0, 95_KX914482_1_FD5-5:0):0, 96_KX914479_1_FD5-8:0):0.0014, ((81_KX914590_1_EpD5-17:0, 82_KX914587_1_EpD5-21:0):0, 97_KX914478_1_FD5-9:0):0.0014):0.0021, 78_KX914602_1_EpD4-23:0.00349):0.00111, (((((67_KX914635_1_EpD2-20:0, 70_KX914631_1_EpD2-3:0):0, 72_KX914623_1_EpD3-13:0):0, 73_KX914618_1_EpD3-5:0):0, 74_KX914616_1_EpD3-7:0):0, 83_KX914558_1_EpS4-4:0):0.0046):0.00195, 85_KX914530_1_FD2-13:0.00655):0.00365, 84_KX914545_1_EpS5-9:0.01021):0.00283, 75_KX914615_1_EpD3-8:0.01304):0.00355, (69_KX914632_1_EpD2-2:0.00849, 79_KX914595_1_EpD5-10:0.00849):0.0081):0.00872, (((93_KX914488_1_FD5-20:0.0131, 80_KX914594_1_EpD5-11:0.0131):0.00333, 77_KX914603_1_EPD4-22:0.01643):0.0042, 183_JX842791_1_SCE12:0.02063):0.00468):0.00956, (147_JX842842_1_SCE3:0.02454, 163_JX842815_1_SEE12:0.02454):0.01032):0.00488, 165_JX842812_1_SEE9:0.03975):0.00771):0.00183, (((155_JX842829_1_SNOE2:0.01999, 195_JX842774_1_SC5:0.01999):0.00267, 170_JX842807_1_SEE4:0.02266):0.0206, (176_JX842800_1_SE9_2012_:0.03709, 166_JX842811_1_SEE8:0.03709):0.00617):0.00603):0.00704, (149_JX842837_1_SNOE10:0.03637, 188_JX842786_1_SCE7:0.03637):0.01996):0.00445, (((((193_JX842780_1_SC11:0.01866, 186_JX842788_1_SCE9:0.01866):0.00533, 197_JX842772_1_SC2:0.02399):0.00998, ((150_JX842836_1_SNOE9:0.01692, 159_JX842821_1_SNO6:0.01692):0.00797, 164_JX842813_1_SEE10:0.0249):0.00908):0.00717, (161_JX842818_1_SNO3:0.02515, 162_JX842817_1_SNO2:0.02515):0.01599):0.00421, (110_KC493319_1_SPCSM55:0.03571, 167_JX842810_1_SEE7:0.03571):0.00964):0.00798, 184_JX842790_1_SCE11:0.05334):0.00744):0.00864, (((20_MN121763_1_2_7A:0.02597, (114_KC871627_1_SPEL35B:0.01814, 160_JX842820_1_SNO5:0.01814):0.00783):0.01078, 172_JX842804_1_SEE1:0.03675):0.02239, 128_KC867323_1_SSO7_1:0.05914):0.01028):0.01174):0.01779, (((129_KC859435_1_SSO5_1:0.06131, ((((((24_MK424606_1_ESA_405:0.01647, 53_MF442293_1_ERR_771:0.01647):0.0016, 27_MK424598_1_ESA_397:0.01807):0.00693, 144_JX174203_1_S2326:0.025):0.00238, (60_KX884918_1_1S:0.02189, 143_JX174222_1_S2345:0.02189):0.00549):0.0062, 28_MK424592_1_ESA_391:0.03358):0.0091, (((50_MF442301_1_ERR_750:0.01483, ((52_MF442294_1_ERR_770:0.0089, 54_MF442289_1_ERR_782:0.0089):0.00268, (57_MF442268_1_ERR_849:0.00479, 58_MF442264_1_ERR_873:0.00479):0.00679):0.00325):0.00844, (4_OM912648_1_CNPF_179:0.00843, 25_MK424604_1_ESA_403:0.00843):0.01484):0.01707, 56_MF442269_1_ERR_843:0.04034):0.00234):0.01863):0.02555, (23_MK424608_1_ESA_407:0.03319, (117_KJ524243_1_Shata69:0.00881, 122_KJ524171_1_Sacu68:0.00881):0.02438):0.05367):0.00617, 204_FJ748679_1_SICB130:0.09303):0.00592):0.02681, 168_JX842809_1_SEE6:0.12577):0.00415, 137_JF488016_1_SWr1C11:0.12991):0.01371, ((((22_MK424612_1_ESA_411:0.07837, (175_JX842801_1_SE10:0.06899, 169_JX842808_1_SEE5:0.06899):0.00938):0.03038, ((148_JX842838_1_SNOE11:0.0636, (157_JX842825_1_SNO10:0.05048, 180_JX842795_1_SE4:0.05048):0.01312):0.03862, 178_JX842797_1_SE6:0.10222):0.00653):0.02599, ((158_JX842823_1_SNO8:0.08759, 194_JX842777_1_SC8:0.08759):0.04576, 196_JX842773_1_SC3:0.13335):0.0014):0.00143, (205_FJ748678_1_SICB133:0.10286, 185_JX842789_1_SCE10:0.10286):0.03332):0.00744):0.01296, ((17_MK942823_1_B30_03:0.04983, 18_MK942822_1_B30_02:0.04983):0.03872, (16_MK942824_1_A25_02:0.04922, 55_MF442273_1_ERR_828:0.04922):0.03932):0.06804):0.04428, ((((((((3_OP810516_1_C7-2C:0.01208, 43_MF326458_1_118L:0.01208):0.0049, (45_MG685722_1_COD59:0.01348, 59_KX884938_1_21S:0.01348):0.00349):0.017, 12_OL631162_1_CMA26:0.03397):0.02228, (19_MN122077_1_2_30S:0.03504, 49_MF442343_1_ERR_859:0.03504):0.02121):0.01025, ((13_MW139990_1_ICBG1883:0.01986, (14_MW139989_1_ICBG1882:0.01432, 15_MW139985_1_ICBG1877:0.01432):0.00554):0.02407, ((10_OM688833_1_B734/16:0.02183, 21_MK649681_1_UFLA01-759:0.02183):0.00794, 48_MF442344_1_ERR_640:0.02977):0.01416):0.02257):0.02065, (209_FJ748674_1_SICB209:0.05417, 199_JF303694_1_S5PR:0.05417):0.03298):0.01808, (198_JF303702_1_S7SC:0.06944, 127_KC867327_1_SSO7_5:0.06944):0.03579):0.02772, 208_FJ748675_1_SICB194:0.13296):0.03074, 5_OM912645_1_CNPF_277:0.1637):0.03716):0.04494, (203_FJ748680_1_SICB129:0.20169, 102_KX170975_1_Uncultured_Sclone_Cu_86:0.20169):0.04411):0.07091, 99_KX856236_1_LMA62:0.31671):0.09156, ((((130_KF496110_1_SBac31D2:0.13012, 153_JX842832_1_SNOE5:0.13012):0.10646, 192_JX842781_1_SCE1:0.23658):0.05741, 113_KP036892_1_SDsA_N007:0.29399):0.0682, 202_FJ748681_1_SICB128:0.3622):0.04607):0.08459, ((((34_MF495732_1_UFLA03-682:0.15306, 47_KX618354_1_R218:0.15306):0.08115, 156_JX842826_1_SNO11:0.23421):0.0203, 173_JX842803_1_SE12:0.25451):0.20119, 98_KX856261_1_LMA87:0.4557):0.03716):0.1582, 187_JX842787_1_SCE8:0.65106):0.29351):0.16492, (29_KY229232_1_UNVERIFIED__UAGC_982:0.12142,

	30_KY229231_1_UNVERIFIED__UAGC_965:0.12142):0.98808):0.11022, ((212_KT970697_1_Lysinibacillus_sp__6P:0.00986, 213_KT962906_1_Lysinibacillus_sp__13P:0.00986):0.05737, 214_KT962904_1_Bacillus_sp__10G:0.06723):1.15249):0.14178, 65_KY040133_1_A37:1.36149);<br><br>end;
